# ER Adaptor SCAP Translocates and Recruits IRF3 to Perinuclear Microsome Induced by Cytosolic Microbial DNAs

**DOI:** 10.1371/journal.ppat.1005462

**Published:** 2016-02-22

**Authors:** Wei Chen, Senlin Li, Huansha Yu, Xing Liu, Lulu Huang, Qiang Wang, Heng Liu, Ye Cui, Yijun Tang, Peng Zhang, Chen Wang

**Affiliations:** 1 State Key Laboratory of Cell Biology, CAS Center for Excellence in Molecular Cell Science, Innovation Center for Cell Signaling Network, Institute of Biochemistry and Cell Biology, Shanghai Institutes for Biological Sciences, Chinese Academy of Sciences, Shanghai, China; 2 Key Laboratory of Medical Reprogramming Technology, Shenzhen Second People’s Hospital, First Affiliated Hospital of Shenzhen University, Shenzhen, China; 3 Department of Thoracic Surgery, Shanghai Pulmonary Hospital, Tongji University, School of Medicine, Shanghai, China; University of Southern California, UNITED STATES

## Abstract

Stimulator of interferon genes (STING, also known as MITA, ERIS or MPYS) induces the activation of TBK1 kinase and IRF3 transcription factor, upon sensing of microbial DNAs. How IRF3 is recruited onto the STING signalosome remains unknown. We report here that silencing of the ER adaptor SCAP markedly impairs the IRF3-responsive gene expression induced by STING. *Scap* knockdown mice are more susceptible to HSV-1 infection. Interestingly, SCAP translocates from ER, via Golgi, to perinuclear microsome in a STING-dependent manner. Mechanistically, the N-terminal transmembrane domain of SCAP interacts with STING, and the C-terminal cytosolic domain of SCAP binds to IRF3, thus recruiting IRF3 onto STING signalosome. Mis-localization of SCAP abolishes its antiviral function. Collectively, this study characterizes SCAP as an essential adaptor in the STING signaling pathway, uncovering a critical missing link in DNAs-triggered host antiviral responses.

## Author Summary

The stimulator of interferon genes (STING/MITA/ERIS/MPYS) is characterized as the converging point of the cytosolic DNAs-triggered innate immune signaling, and its function has been well documented in mediating the production of type I interferon and other pro-inflammatory cytokines. It remains intriguing to address how IRF3 is recruited onto the STING signalosome. In this study, we have further identified and characterized the SREBP cleavage-activating protein (SCAP) as the long-sought-after adaptor of the STING signaling. Upon microbial DNA challenge, SCAP translocates from ER, via Golgi, to perinuclear microsome in a STING-dependent manner. SCAP thus serves as a scaffold adaptor to recruit IRF3 and facilitate its integration into the perinuclear microsomes. Our study reveals an important missing link in innate immunity, further highlighting the physical and/or functional links between innate immunity and metabolism.

## Introduction

Microbial infections represent an ever-present threat to host homeostasis and survival. The extracellular and intracellular microbes are dynamically and rapidly sensed by specific Pattern Recognition Receptors (PRRs), including TLRs, NLRs and RLRs [1–3]. Upon recognition of the conserved Pathogen Associated Molecular Patterns (PAMPs), PRRs initiate a myriad of signal transduction pathways, triggering innate and adaptive immune responses to eliminate the microbial pathogens [4,5].

DNAs derived from DNA viruses, bacteria or damaged host cells could activate the IRF3 and/or NF-κB signaling pathways, thus inducing the production of type I interferons (IFNs) and other pro-inflammatory cytokines [6,7]. How cells sense and respond to RNA virus infection is well characterized in the past decade [8–10]. Our understanding of the DNA-triggered signaling is relatively limited. TLR9 detects CpG DNA from endolysosome in the immune cells [11]. Multiple cytosolic sensors are proposed to detect viral or microbial DNAs in cytosol, including cGAS, RNA polymerase III, Mre11, DNA-PKcs, IFI16 and DDX41 [12–18]. Further studies are needed to clarify the physiological relevance of some of the putative DNA sensors, and to address the biochemical and functional interactions among these sensors.

Stimulator of interferon genes (STING, also known as MITA, ERIS or MPYS) is characterized as the converging point of the recently identified DNA sensors. STING is an Endoplasmic Reticulum (ER)-associated membrane protein, indispensable for inducing the antiviral innate responses triggered by microbial DNAs [19–22]. For examples, STING-deficient cells fail to induce type I IFN production after stimulation of dsDNA or infection with herpes simplex virus 1 (HSV-1) or *Listeria monocytogenes* [23]. STING knockout mice are highly susceptible to lethal infection by HSV-1 [23]. STING can also bind directly to cyclic dinucleotide (CDNs), including cGAMP, c-di-GMP and c-di-AMP [24,25].

CDNs and/or upstream DNA sensors could induce STING dimerization, causing its translocation from the ER, via Golgi, to perinuclear microsome [21,23,26]. Recently, we have identified the unexpected function of the autocrine motility factor receptor (AMFR, a.k.a GP78) and the insulin induced gene 1 (INSIG1) in innate immunity [27]. AMFR and INSIG1 are ER-resident ubiquitin E3 ligase, responsible for catalyzing the K48-linked poly-ubiquitination of the ER misfolded proteins, a process essential for the ER Associated Degradation (ERAD) [28]. We characterize AMFR/INSIG1 to interact specifically with STING, and to catalyze the K27-linked poly-ubiquitination of STING. The K27-linked polyubiquitin chain on STING serves as an anchoring platform for recruiting and activating TBK1, which then phosphorylates the transcription factor IRF3 [27]. Notably, IRF3 could not bind to the K27- or K63- linked polyubiquitin chain. How IRF3 is recruited onto the STING signalosome remains largely unknown.

SREBP cleavage-activating protein (SCAP) is a polytopic membrane protein on ER, harboring an N-terminal domain with eight transmembrane helices, and a C-terminal domain with five WD-repeat [29]. It is well established that SCAP interacts with INSIG1 and modulates the lipid homeostasis [30]. In this study, we demonstrate that SCAP interacts with STING independent of INSIG1, and SCAP is indispensable for DNAs-triggered host antiviral responses. Upon HSV-1 infection, SCAP translocates from ER, via Golgi, to perinuclear microsome in a STING-dependent manner. SCAP thus serves as a scaffold adaptor to recruit IRF3 onto the STING signalosome, which reveals a critical missing link in innate immunity.

## Results

### Identification of SCAP in the STING signalosome

**Fig 1:**
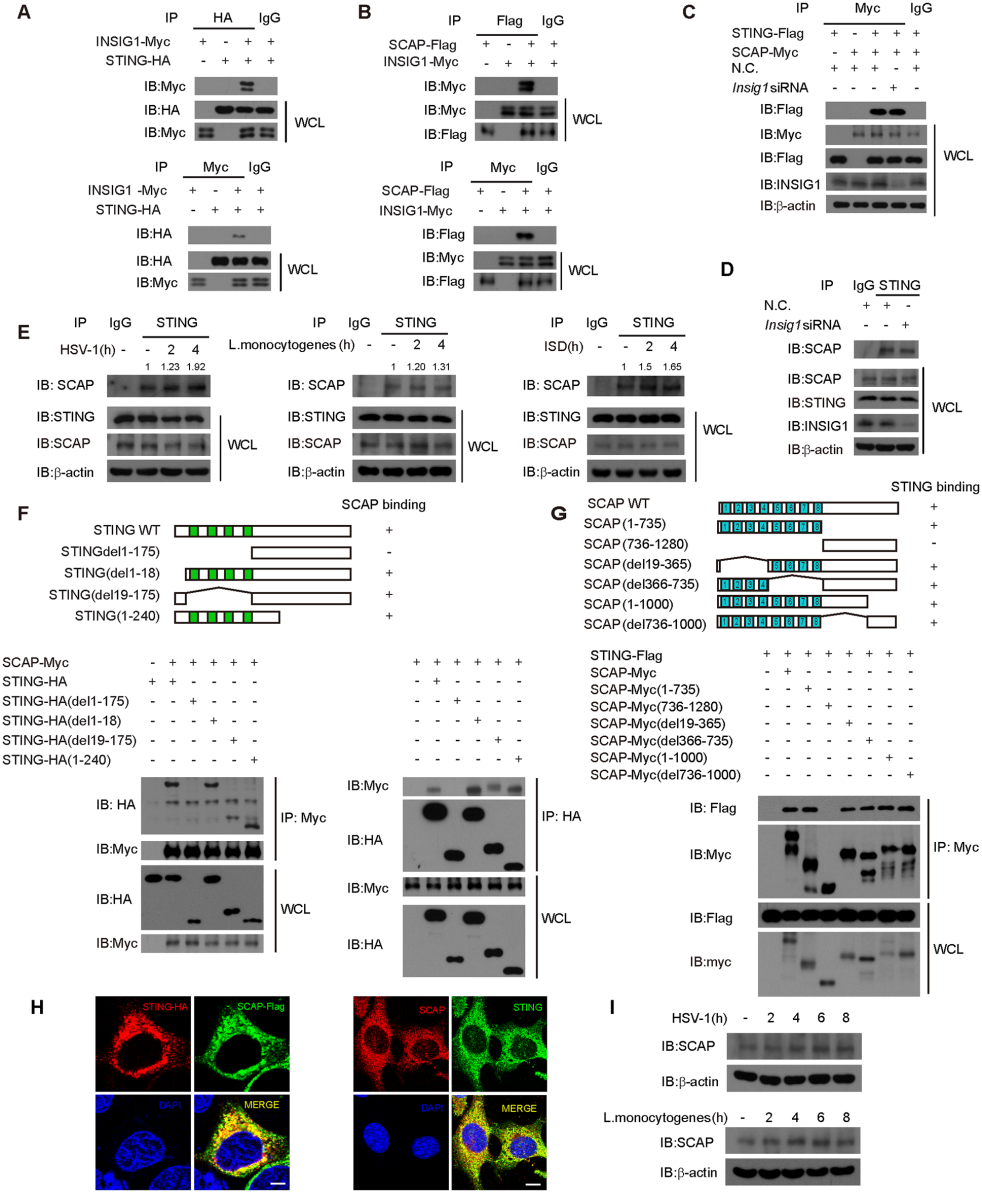
Identification of SCAP in STING protein complex. (A and B) HEK293T cells were transfected with indicated plasmids. Twenty-four hours after transfection, cell lysates were immunoprecipitated with an anti-HA antibody (A, upper), an anti-Flag antibody (B, upper) or an anti-Myc antibody (A and B, lower), and then immunoblotted with indicated antibodies. The normal IgG served as negative control. (C) MEF cells were transfected with nonspecific control (N.C.) or *Insig1* siRNA. Twenty-four hours later, STING-Flag and SCAP-Myc were transfected into the knockdown cells. Cell lysates were subjected to immunoprecipitation with an anti-Myc antibody or normal IgG, and then immunoblotted with indicated antibodies. (D) MEF cells were transfected with N.C. or *Insig1* siRNA. Forty-eight hours later, cell lysates were subjected to immunoprecipitation with an anti-STING antibody or normal IgG, and then immunoblotted with indicated antibodies. (E) After stimulation with HSV-1 (left), *Listeria monocytogenes* (middle) or ISD (right) in indicated time periods, lysates of MEF cells were immunoprecipitated with an anti-STING antibody or normal IgG, and then immunoblotted with indicated antibodies. (F) Schematic diagram of STING and its truncation mutants (upper panel). HA-tagged STING or its mutants were individually transfected into HEK293T cells along with Myc-tagged SCAP. The cell lysates were immunoprecipitated with an anti-Myc antibody (left) or an anti-HA antibody (right) and then immunoblotted with indicated antibodies.(G) Schematic diagram of SCAP and its truncation mutants (upper panel). Myc-tagged SCAP or its mutants were individually transfected into HEK293T cells along with Flag-tagged STING. The cell lysates were immunoprecipitated with an anti-Myc antibody and then immunoblotted with indicated antibodies (lower panel). (H) Hela cells were transfected with HA-tagged STING and Flag-tagged SCAP, and then stained with indicated antibodies before imaged by confocal microscopy (left). MEF cells were immunostained with indicated antibodies and imaged by confocal microscopy (right). Scale bars represent 10μm. (I) MEF cells were infected with HSV-1 (upper) or *Listeria monocytogenes* (lower) for the indicated time periods, and then the cell lysates were immunoblotted with an anti-SCAP antibody. β-actin was served as loading control.

Our recent study [27] identified INSIG1 to specifically interact with STING (Fig 1A). Given that INSIG1 interacts with SCAP in lipid metabolism [30], we wondered whether SCAP was also a component in the STING signalosome. We confirmed the association between INSIG1 and SCAP via the co-immunoprecipitation assay (Fig 1B). We observed that SCAP associated with STING exogenously and endogenously (Fig 1C and 1D). This association was marginally enhanced upon HSV-1 (Fig 1E left), *Listeria monocytogenes* (Fig 1E middle) or ISD (Fig 1E right) stimulation. Unexpectedly, silencing of INSIG1 did not affect the association between STING and SCAP (Fig 1C and 1D), indicating that STING associates with SCAP in an INSIG1-independent manner. This suggests that the STING signalosome is physically and functionally distinct from the Lipid Regulatory Complex.

The transmembrane region of STING (1–175 aa) was important for its interaction with SCAP (Fig 1F). Likewise, the transmembrane region of SCAP (1–735 aa) was mapped to mediate the same interaction (Fig 1G). Confocal microscope imaging confirmed that SCAP co-localized with STING exogenously and endogenously (Fig 1H). HSV-1 and *Listeria monocytogenes* infections marginally induced the expression of SCAP (Fig 1I). Taken together, these data suggest that SCAP is a new component in the STING protein complex.

### Silencing of *Scap* attenuates cytosolic DNA-induced IRF3 activation

**Fig 2:**
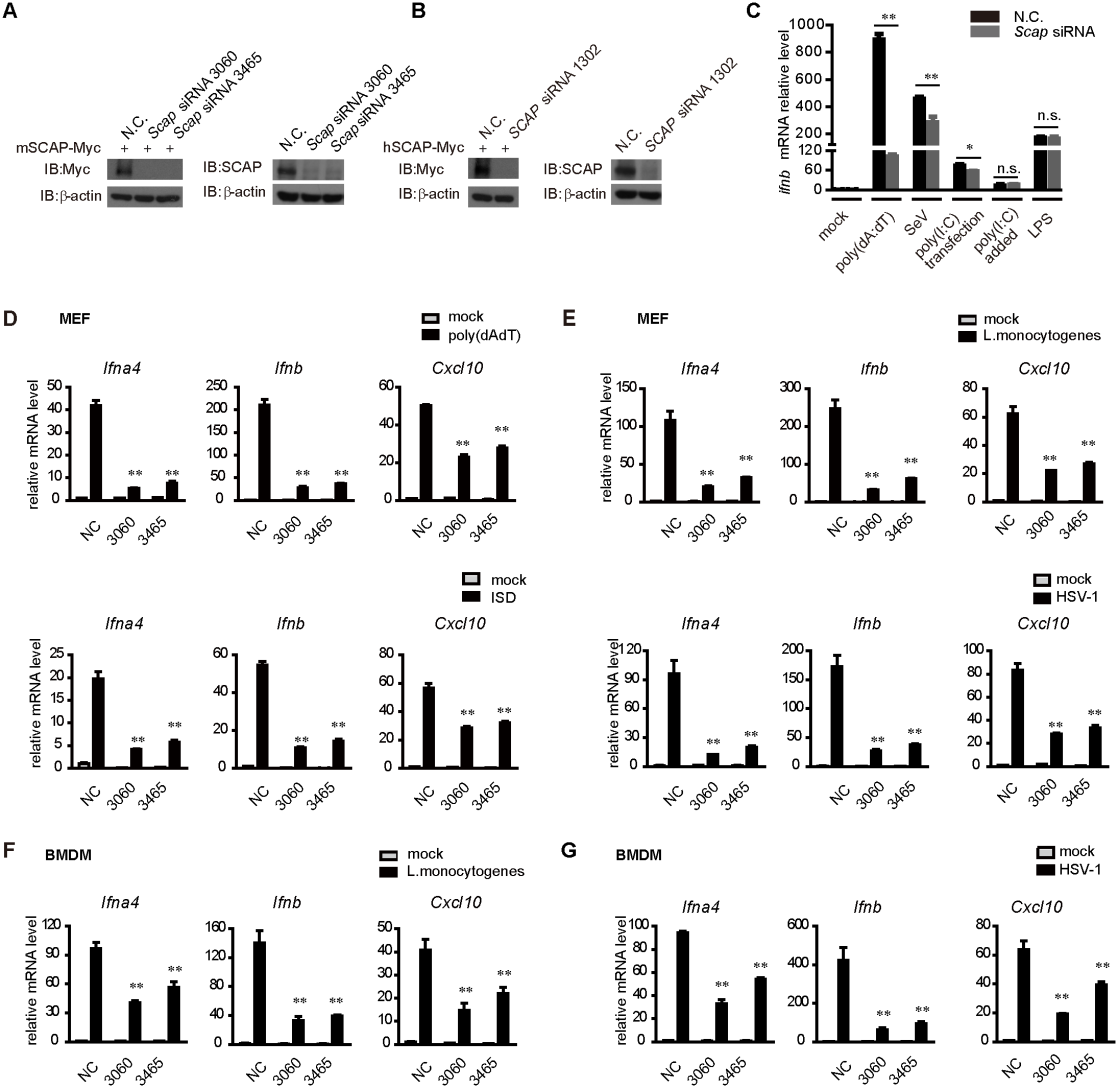
Silencing of *Scap* inhibits intracellular DNA-mediated IRF3 activation. (A) HEK293T cells were transfected with Myc-tagged mouse SCAP and then treated with the nonspecific control (N.C.) or *Scap* siRNAs (left panel). MEF cells were transfected with the negative control (N.C.) or *Scap* siRNAs (right panel). Cell lysates were immunoblotted with the indicated antibodies. (B) HEK293T cells were transfected with Myc-tagged human SCAP and then treated with the nonspecific control (N.C.) or *SCAP* siRNA (left panel). HEK293T cells were transfected with the negative control (N.C.) or *SCAP* siRNA (right panel). Cell lysates were immunoblotted with the indicated antibodies. (C) The nonspecific control (N.C.) or *Scap* siRNA were transfected into MEF cells. Forty-eight hours after transfection, cells were stimulated with poly(dA:dT), SeV, poly(I:C) (transfected), poly(I:C) (added to the culture medium) or LPS. Induction of *Ifnb* mRNA was measured by quantitative PCR. (D and E) The indicated siRNAs were transfected into MEF cells. Induction of *Ifna4*, *Ifnb*, and *Cxcl10* mRNAs was measured by quantitative PCR after poly(dA:dT) (D, upper) or ISD (D, lower) stimulation, *Listeria monocytogenes* infection (E, upper) or HSV-1 invasion (E, lower). (F and G) The indicated siRNAs were transfected into BMDMs. Induction of *Ifna4*, *Ifnb*, and *Cxcl10* mRNA was measured by quantitative PCR after *Listeria monocytogenes* (F) or HSV-1 (G) infection. Data from (C-G) are presented as means ± SD from three independent experiments. *p < 0.05; **p < 0.01.

To probe the potential function of SCAP in innate immunity, we screened out the specific and effective siRNAs against *Scap* (*Scap* siRNA 3060, *Scap* siRNA 3465 for mouse and *SCAP* siRNA 1302 for human), all of which could markedly diminish the expression of exogenous and endogenous SCAP (Fig 2A and 2B). It was observed that knockdown of endogenous *Scap* inhibited the DNA mimics poly(dA:dT)-triggered induction of *Ifnb* mRNA (Fig 2C). In contrast, poly(I:C)- or SeV-triggered RIG-I signaling was marginally affected in *Scap* knockdown cells (Fig 2C). Knockdown of *Scap* had no inhibitory effects on the TLRs-mediated activation of the *Ifnb* mRNA triggered by LPS or poly(I:C) added (Fig 2C). These data indicate that SCAP specifically regulates the cytosolic DNA-triggered expression of IFN-β.

To substantiate, we explored the effect of *Scap* knockdown on the expression of IRF3-responsive genes induced by cytosolic DNA challenge, using qPCR (quantitative PCR). As expected, silencing of *Scap* markedly attenuated the induction of the IRF3-responsive genes (*Ifnb*, *Ifna4* and *Cxcl10*) in MEF cells, stimulated by the DNA mimics [poly(dA:dT) or ISD] (Fig 2D) treatment or the DNA pathogens (*Listeria monocytogenes* or HSV-1) (Fig 2E). However, silencing of SCAP apparently displayed no effect on the Thapsigargin-induced ER stress (S1 Fig).

To make it more physiologically relevant, we further investigated the function of SCAP in primary cells. BMDMs (bone marrow derived macrophage) were transfected with siRNAs against *Scap*, followed by HSV-1 or *Listeria monocytogenes* stimulation. Consistently, silencing of *Scap* markedly attenuated the expression of IRF3-responsive genes in BMDMs (Fig 2F and 2G). Collectively, these data indicate that SCAP is a positive modulator of the cytosolic DNA-triggered STING signaling pathway.

### SCAP functions downstream of STING and upstream of IRF3

**Fig 3:**
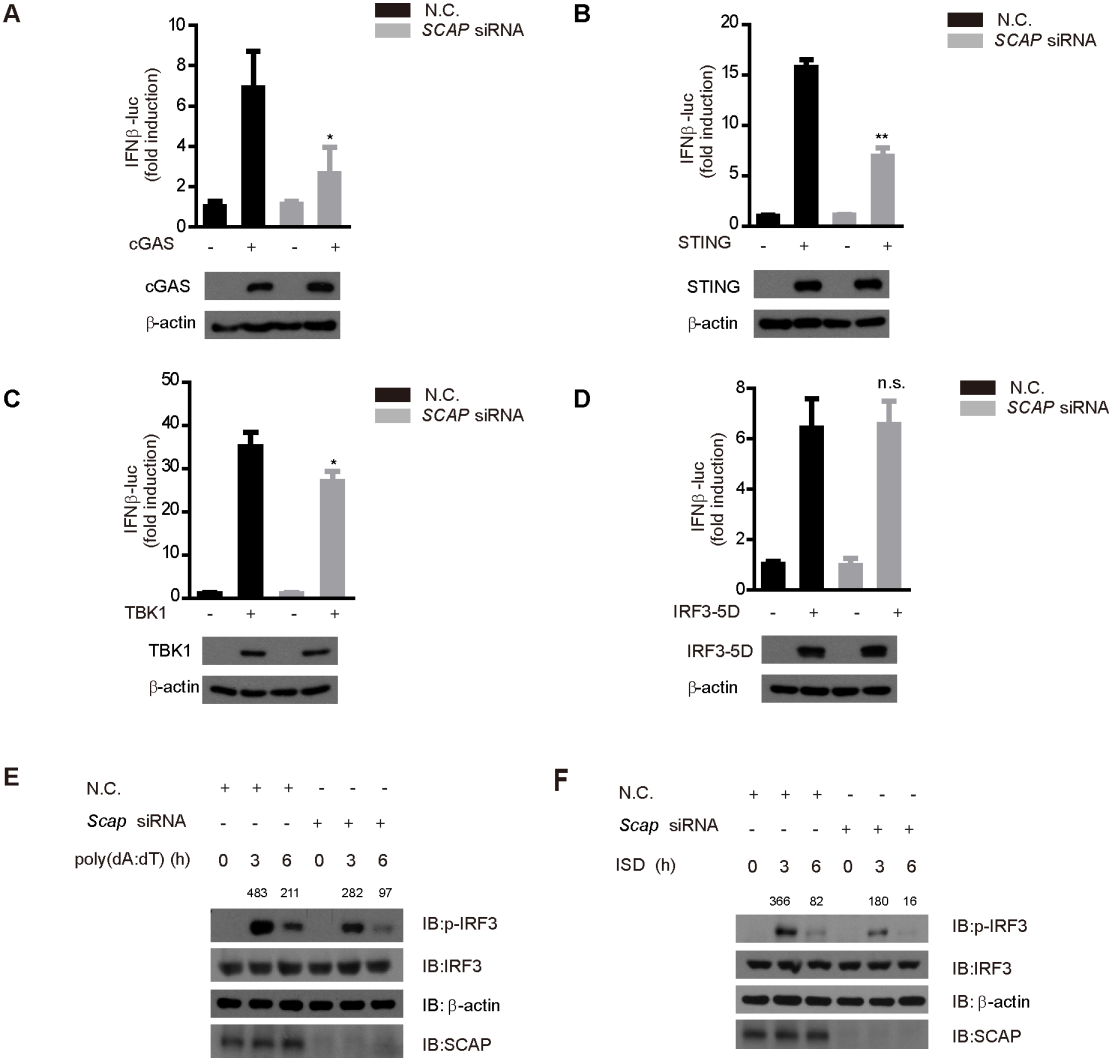
SCAP functions at downstream of STING and upstream of IRF3. (A-D) The indicated siRNA were transfected into HEK293 cells together with IFN-β-luciferase and pTK-Renilla reporter plasmids. Twenty-four hours after transfection, cells were transfected again with cGAS (A), STING (B), TBK1 (C) or IRF3-5D (D) for twenty-four hours before luciferase assays were performed. (E and F) The nonspecific control (N.C.) or *Scap* siRNA were transfected into MEF cells. Forty-eight hours after transfection, cells were stimulated with poly(dA:dT) (E) or ISD (F) for indicated time periods, and cell extracts were analyzed for IRF3 phosphorylation. Data from (A-D) are presented as means ± SD from three independent experiments. *p < 0.05; **p < 0.01.

We further delineated the topology of SCAP in the STING signaling pathway. Exogenous expression of cGAS or STING could respectively activate the IFN-β-luciferase reporters, and these activations were obviously impaired when knocking down *Scap* (Fig 3A and 3B). In contrast, knockdown of *Scap* marginally affected the expression of IFN-β-luciferase reporter when ectopically expressing TBK1 (Fig 3C). Likewise, *Scap* knockdown had no effect on the activation of the IFN-β-luciferase reporter, when cells were stimulated with the exogenous IRF3-5D (Fig 3D). Furthermore, ectopic expression of SCAP only or both SCAP and INSIG1 could not activate the IFN-β-luciferase reporter (S2A and S2B Fig). Given the hierarchical relationships among these signaling molecules, we reasoned that SCAP modulates the STING signaling downstream of STING and upstream of IRF3.

Consistently, the expressions of PRDIII-I-luciferase reporters stimulated by cGAS, STING or TBK1 were attenuated in *Scap* knockdown cells, whereas the expressions of PRDIII-I-luciferase reporters stimulated by IRF3-5D remained intact in *Scap* knockdown cells (S3A Fig). The ubiquitination of STING or the recruitment of TBK1 was not affected by endogenous SCAP depletion (S3D to S3G Fig). Notably, knockdown of *Scap* led to an apparent decrease in the phosphorylation of IRF3, but not that of TBK1, when stimulating cells with either poly(dA:dT) (Fig 3E and S3B Fig) or ISD (Fig 3F and S3C Fig). Consistently, ISD-triggered dimerization and nuclear translocation of IRF3 were markedly impaired when silencing SCAP. In contrast, knockdown of Tom20 could not influence the dimerization and nuclear translocation of IRF3 (S4A and S4B Fig).

### SCAP translocates to perinuclear microsome in a STING-dependent manner

**Fig 4:**
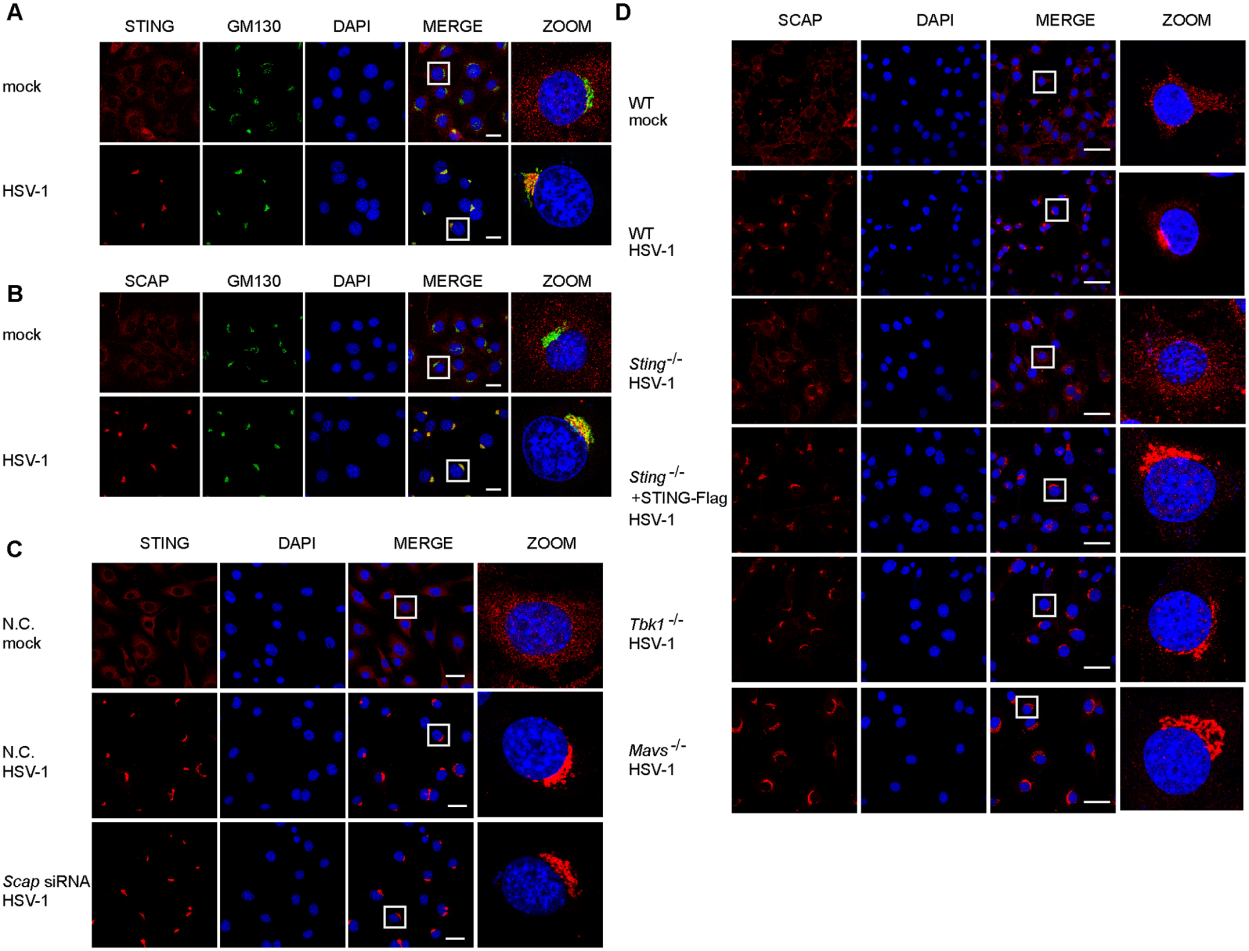
SCAP translocates to perinuclear microsome in a STING-dependent manner. (A and B) Immunofluorescence microscopy of STING (A) or SCAP (B) in MEFs infected with or without HSV-1. The Golgi was stained with GM130. Scale bars represent 25μm. (C) MEF cells were transfected with N.C. or *Scap* siRNA. After stimulation with HSV-1, MEF cells were immunostained with an anti-STING antibody and imaged by confocal microscopy. Scale bars represent 25μm. (D) WT or indicated deficient MEF cells treated with HSV-1 were immunostained with an anti-SCAP antibody and imaged by confocal microscopy. Scale bars represent 50μm.

We confirmed via confocal microscopy that STING traffics from the ER to perinuclear/Golgi foci (also called perinuclear microsome) upon HSV-1 infection (Fig 4A). We wondered whether this translocation was modulated by SCAP. This possibility was ruled out by the observation that the STING translocation was intact when silencing *Scap* (Fig 4C). Unexpectedly, HSV-1 infection also triggered SCAP to translocate from ER to the perinuclear microsome (Fig 4B). The Cell fractionation assay further revealed that HSV-1 infection induced both STING and SCAP to be predominantly in the microsome fraction (S5C Fig). Notably, SCAP was co-localized with STING both before and after the HSV-1 infection (Fig 1H and S5A Fig). We reasoned that the SCAP translocation is instead dependent on STING.

To test this hypothesis, we monitored the SCAP aggregation in wild-type and *Sting* ^-/-^ MEFs upon HSV-1 infection. As expected, SCAP congregated to perinuclear microsome in wild-type MEFs upon HSV-1 infection (Fig 4D). Interestingly, STING deficiency dramatically reduced the trafficking of SCAP to the perinuclear foci (Fig 4D). This translocation of SCAP was obviously rescued in the *Sting* ^-/-^ MEFs reconstituted with Flag-tagged STING (Fig 4D). In *Mavs* ^-/-^ or *Tbk1* ^-/-^ MEFs, SCAP could also congregate to perinuclear microsome (Fig 4D). Taken together, these data indicate that STING specifically facilitates the translocation of SCAP to the perinuclear microsome.

### SCAP recruits IRF3 onto the STING signalosome

**Fig 5:**
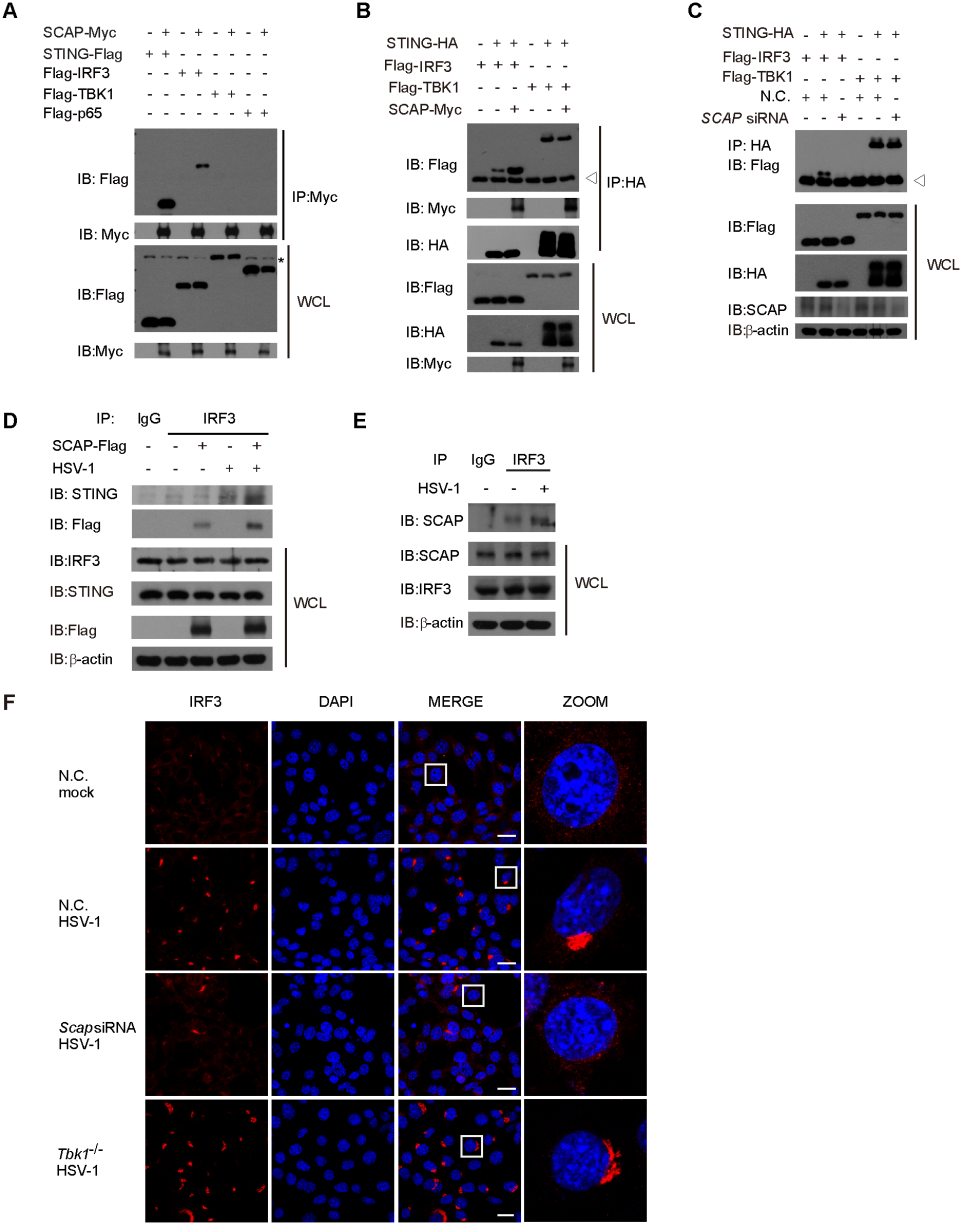
SCAP recruits IRF3 onto the STING signalosome. (A) The indicated plasmids with Flag tag were respectively transfected into HEK293T cells along with Myc-tagged SCAP. Cell lysates were immunoprecipitated with an anti-Myc antibody and immunoblotted with indicated antibodies. A nonspecific band is indicated by an asterisk. (B) HEK293T cells were transfected with indicated plasmids. The cell lysates were immunoprecipitated with an anti-HA antibody and immunoblotted with indicated antibodies. lgG band is indicated by a triangle. (C) HEK293T cells were transfected with N.C. or *SCAP* siRNA. Twenty-four hours later, indicated plasmids were transfected into the knockdown cells. Cell lysates were subjected to immunoprecipitation with an anti-HA antibody and immunoblotted with the indicated antibodies. lgG band is indicated by a triangle. (D) MEF cells with or without stabilized overexpression of Flag-tagged SCAP was infected with HSV-1, cell lysates were immunoprecipitated with an anti-IRF3 antibody and immunoblotted with indicated antibodies. (E) MEF cells were infected with HSV-1, cell lysates were immunoprecipitated with an anti-IRF3 antibody and immunoblotted with indicated antibodies. (F) WT MEF cells transfected with N.C. or *Scap* siRNA for forty-eight hours, or TBK1 deficient MEF cells were pretreated with the TBK1 kinase inhibitor BX795 and then stimulated with HSV-1, then cells were immunostainned with an anti-IRF3 antibody and imaged by confocal microscopy. Scale bars represent 25μm.

To explore the action of SCAP, we noticed that SCAP interacted strongly with both STING and IRF3, but not with TBK1 and p65 (Fig 5A). The C-terminal cytosolic domain of SCAP (736–1280 aa) was mapped to bind to IRF3 (201–357 aa) (S6A and S6B Fig), whereas the transmembrane region of SCAP (1–735 aa) was mediated to interact with STING (Fig 1G). So we speculated that SCAP might serve as an adaptor for recruiting IRF3 onto STING. The association of STING and IRF3 was enhanced in the presence of SCAP, whereas silencing of *Scap* almost abolished this association. In contrast, SCAP did not affect the interaction between STING and TBK1 (Fig 5B and 5C). In addition, ectopic-expression of SCAP promoted the endogenous association of STING and IRF3 in response to the HSV-1 stimulation (Fig 5D). Notably, knockdown of SCAP impaired the endogenous association of STING and IRF3 (S6C Fig). The endogenous interaction between SCAP and IRF3 was also confirmed, and this interaction was enhanced upon HSV-1 stimulation (Fig 5E). In *sting*-/- MEFs, the interaction of SCAP and IRF3 is markedly attenuated (S6D Fig), which indicates that SCAP recruits IRF3 after its STING-dependent translocation to the microsomes.

Notably, IRF3 also congregated to the perinuclear microsome and co-localized with SCAP upon HSV-1 infection (Fig 5F and S5B Fig). Importantly, silencing of *Scap* blocked the congregation of IRF3 (Fig 5F), whereas TBK1 deficiency did not affect the trafficking of IRF3 to the perinuclear foci (Fig 5F). Taken together, these data indicate that SCAP bridges STING to IRF3 in the perinuclear microsome.

### Mis-localization of SCAP impairs its antiviral function

**Fig 6:**
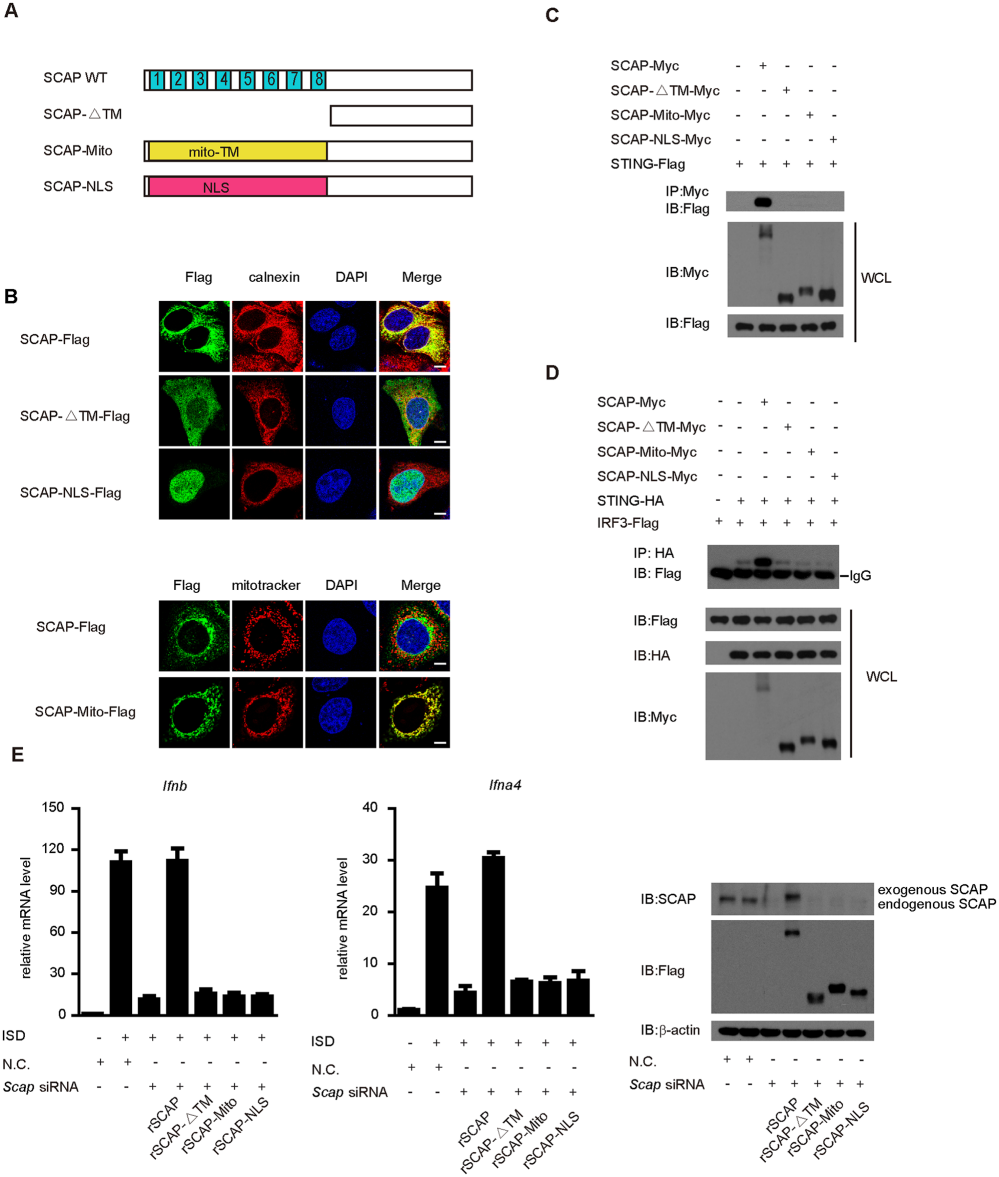
Mis-localization of SCAP impairs its antiviral function. (A) Schematic diagram of wild-type SCAP and its mis-localization mutants. (B) The indicated constructs were individually transfected into Hela cells and then stained with an anti-Flag antibody before imaged by confocal microscopy. The ER was stained with calnexin (upper panel). MEF cells transfected with the indicated plasmids were stained with an anti-Flag antibody and imaged by confocal microscopy. The mitochondria were stained with MitoTracker (lower panel). Scale bars represent 10μm. (C) Myc-tagged SCAP or its mutants was transfected into HEK293T cells along with Flag-tagged STING. Twenty-four hours after transfection, cell lysates were immunoprecipitated with an anti-Myc antibody, and then immunoblotted with the indicated antibodies. (D) STING-HA and Flag-IRF3 were transfected into HEK293T cells along with Myc tagged SCAP or SCAP mutants, respectively. Cell lysates were subjected to immunoprecipitation with an anti-HA antibody, and then immunoblotted with the indicated antibodies. (E) MEF cells were transfected with N.C. or *Scap* siRNA and then rescued with the indicated siRNA-resistant SCAP constructs. After ISD stimulation, induction of *Ifnb* and *Ifna4* mRNA was measured by qPCR. Data are presented as means ± SD from three independent experiments.

To determine the importance of ER localization for SCAP function, we generated three mis-localization mutants of SCAP. SCAP-ΔTM was constructed by deleting the N-terminal transmembrane domain of SCAP. SCAP-Mito was constructed by replacing the transmembrane domain with the mitochondria targeting sequence from Tom70 (translocase of outer membrane 70, a mitochondria membrane protein). SCAP-NLS was constructed by replacing the transmembrane domain with a nuclear localization sequence (Fig 6A). Confocal microscopy analysis confirmed that SCAP-ΔTM, SCAP-Mito and SCAP-NLS were targeted to whole cell, mitochondria and nucleus, respectively (Fig 6B). As expected, SCAP-ΔTM, SCAP-Mito and SCAP-NLS failed to interact with STING (Fig 6C). SCAP-NLS also failed to interact with IRF3 (S7 Fig). Notably, SCAP mis-localization mutants could not enhance the association of STING and IRF3 (Fig 6D).

We further performed rescue experiments to corroborate the functional consequences. MEF cells were first transfected with control or *Scap* siRNAs, followed by transfection of the control or the RNAi-resistant rSCAP plasmids, respectively. The induction of *Ifnb* or *Ifna4* mRNA was measured after ISD stimulation. Consistently, the induction of *Ifnb* or *Ifna4* mRNA was restored by wild type rSCAP, but not rescued by SCAP mis-localization mutants rSCAP-ΔTM, rSCAP-Mito or rSCAP-NLS (Fig 6E). Taken together, these data indicate that the ER localization of SCAP is essential for its action in the STING signaling.

### SCAP is indispensable for innate antimicrobial responses

**Fig 7:**
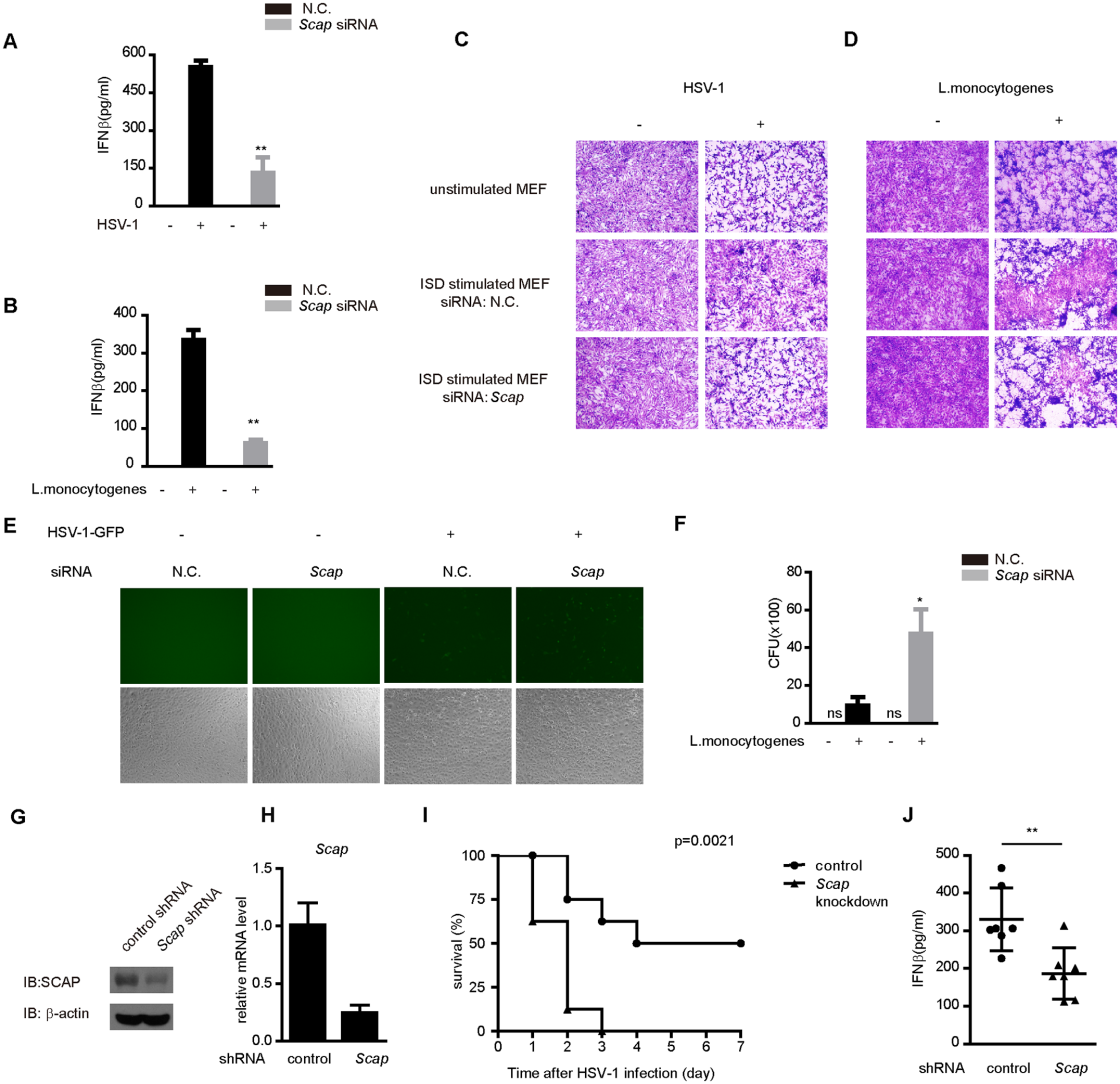
SCAP modulates antimicrobial defenses against HSV-1 and *Listeria monocytogenes*. (A and B) MEF cells were transfected with indicated siRNA. After infection with HSV-1 (MOI = 10) (A) or *Listeria monocytogenes* (B), the supernatants were collected and assayed for IFN-β production by ELISA. (C and D) MEF Cells were transfected with indicated siRNA and then treated with mock or ISD. Equal volumes of culture supernatants from these treatments were applied to fresh MEF cells, followed by HSV-1 (MOI = 10) (C) or *Listeria monocytogenes* (D) infection. The proliferation of cells was examined by crystal violet staining. (E) HSV-1-GFP (MOI = 0.5) replication in MEF cells transfected with N.C. or *Scap* siRNA was visualized by fluorescence microscopy. (F) MEF cells transfected with N.C. or *Scap* siRNA and then were infected with *Listeria monocytogenes*. The supernatant were determined by bacterial colonization assay. (G) Immunoblot analysis of SCAP in lysates of liver tissue from mice at forty-eight hours after transfection with *Scap* or control shRNA. (H) mRNA level of SCAP in leukocyte of blood from mice at forty-eight hours after transfection with Scap or control shRNA. (I) WT or *Scap* knockdown mice (n = 8 per group) were injected intravenously with HSV-1 at 1×10^8^ pfu per mouse and the survival rates were monitored for 7 days. (J) WT or *Scap* knockdown mice (n = 7 per group) were injected intravenously with HSV-1 at 1×10^7^ pfu per mouse. Six hours later, sera were collected and the concentration of IFNβ was measured by ELISA. Data are presented as means ± SD. Data from (A, B and F) are presented as means ± SD from three independent experiments. *p < 0.05; **p < 0.01.

We went on to explore the antiviral function of SCAP in innate immunity. The induction of IFN-β is a hallmark of host antiviral responses. *Scap* siRNA was transfected into MEF cells, followed by HSV-1 (Fig 7A) or *Listeria monocytogenes* (Fig 7B) infection. The supernatants were quantified by ELISA (enzyme-linked immunosorbent assay). As expected, knockdown of endogenous *Scap* drastically impaired the IFN-β protein production (Fig 7A and 7B).

Since IFN-β protects host cells against virus infection, we assessed whether SCAP could restrict HSV-1 infection. MEF cells were respectively pretreated with culture supernatants from ISD-stimulated *Scap* knockdown cells or control cells, followed by HSV-1 (Fig 7C) or *Listeria monocytogenes* (Fig 7D) infection. It was observed that, fresh cells pretreated with culture supernatants from *Scap* knockdown MEFs were more permissive to HSV-1 or *Listeria monocytogenes* infection (Fig 7C and 7D).

In addition, we investigated whether SCAP attenuated microbial replications by challenging cells with HSV-1-GFP or *Listeria monocytogenes*. Consistently, knockdown of *Scap* augmented the levels of HSV-1-GFP-positive cells (Fig 7E), and also markedly enhanced the replication of *Listeria monocytogenes* (Fig 7F). In contrast, the replication of NDV-GFP was unaffected by depletion of *Scap* (S8 Fig).

Finally, we investigated the *in vivo* role of SCAP. We delivered into mice, via tail vein injection, the *Scap* specific or control shRNAs coated with polyethyleneimine (PEI). The efficiency of *in vivo* ‘knockdown’ was confirmed (Fig 7G and 7H). Next, mice were infected intravenously with HSV-1, and their survival rates were monitored. As expected, *Scap*-knockdown mice were more susceptible to HSV-1 infection than control mice. All the *Scap*-knockdown mice died within 3 days, whereas 50% of the control mice remained alive until 7 days after HSV-1 infection (Fig 7I). Notably, *Scap* knockdown mice displayed a severer defect in the production of sera IFN-β upon HSV-1 invasion, as compared with the infected control mice (Fig 7J). These data indicate that SCAP is indispensable for protecting mice against HSV-1 infection.

## Discussion

Recent breakthroughs have characterized multiple cytosolic sensors that potentially monitor the cytosolic DNAs (cGAS, IFI16, DDX41, Mre11 and DNA-PKcs). STING is established unambiguously as the converging point of the DNA sensors, to further relay the activation signals on ER [31,32]. Notably, STING is induced to dimerize and traffic from the ER, via Golgi, to perinuclear microsome [6,23]. TBK1 is simultaneously recruited to the same compartment in a STING dependent manner, which then activates the transcription factor IRF3 [23]. It is intriguing to dissect the molecular mechanisms of the DNA-driven assembly of the STING signalosome on either ER or the perinuclear microsome.

Our recent study [27] has characterized the AMFR/INSIG1 protein complex as a novel component of the STING signalosome on ER. The E3 ubiquitin ligase AMFR, bridged by INSIG1, catalyzes the K27-linked polyubiquitination of STING upon microbial DNA challenge. This unique polyubiquitin chain specifically recruits TBK1 and ferries the latter to the perinuclear microsome, along with STING. Notably, IRF3 could bind neither the K27- nor K63- linked polyubiquitin chain. This suggests that IRF3 is recruited onto the STING signalosome via another uncharacterized mechanism.

So we speculated that there might be unknown adaptor protein(s) on ER to perform this function. An analogy drew our attention regarding the translocations of the STING and SREBP. SREBP is a master transcription factor of the lipid and glucose metabolism on ER, which also translocates from ER to Golgi in response to metabolic stimuli [33]. Notably, SCAP is indispensable for chaperoning SREBP to Golgi, and INSIG1 specifically interacts with SCAP to prevent this translocation [30,34]. This inspires us to explore the potential function of SCAP in innate immunity.

Both STING and SCAP reside on ER via their N-terminal transmembrane domains. We confirmed the interaction between SCAP and STING, and demonstrated the transmembrane domains of both proteins to mediate this interaction. Unexpectedly, this interaction is independent of INSIG1, suggesting that the STING signalosome might be physically and functionally distinct from the Lipid Regulatory Complex. To substantiate, there is no obvious difference of IRF3 activation upon HSV-1stimulation, with or without FBS in the cell culture medium (S9F Fig). SREBP1 knockdown did not influence the exogenous DNA-induced IRF3 activation (S9B and S9C Fig). Furthermore, HSV-1 infection could not trigger SREBP1 translocation (S9D Fig). It was well established that the SREBP signaling was dramatically impaired respectively by three SCAP mutants (SCAP-Y234A, SCAP-Y298C, SCAP-D443N) [35,36]. However, the RNAi-resistant rSCAP-Y234A, rSCAP-Y298C, rSCAP-D443N could rescue the induction of IRF3-responsive genes in SCAP knockdown cells, like that of the wild type rSCAP (S9E Fig). Taken together, these observations indicate that the STING signaling is functionally and physically uncoupled from the SREBP signaling.

During revising this manuscript, York *et al* reported that the cell metabolic reprogramming could also induce the expression of IFNβ and ISGs in a STING-dependent manner [37]. Interestingly, some of the data in this paper suggested that SCAP was also important for this induction. However, they did not address how SCAP could potentially modulate the function of STING.

Notably, there are some discrepancies between the two studies concerning the specific effects of SCAP in regulating the induction of IFNβ and ISGs. York’s study was performed mainly in the context of metabolic reprogramming, whereas our study only focused on the microbe-induced activation of the STING signaling. In our experimental setting, we have supplied enough FBS medium to ensure that the cells will not experience metabolic reprogramming even when knocking down SCAP. In contrast, the indicated paper mainly addressed the metabolic reprogramming-induced expression of IFN-β and ISGs, which broadly affects the overall cell metabolism and dramatically influences the basal expression of IFN-β. Although the observation is interesting, we wondered if this cell model directly or indirectly activates the STING signaling, which needs further exploration in future experiments. For example, it is intriguing to address whether perturbing metabolism will lead to the dimerization and translocation of STING to the perinuclear microsome, whether this activation is dependent on cGAS, whether SCAP and IRF3 will congregate in this scenery. We speculate that different stimulating models could potentially lead to the observed discrepancies.

In addition, we extensively employed HSV-1 and *Listeria Monocytogenes* as effective stimuli to address the activation of STING signaling, which are well characterized microbes in the relevant fields. As far as we know, MHV-68 is scarcely employed in elucidating the STING signaling pathway. We do not know why the indicated study did not use HSV-1 or *Listeria Monocytogenes*. However, MHV-68 could potentially trigger the cGAS-STING signaling [38], and it was recently reported that some RNA viruses could also activate IRF3 in a STING-dependent manner [32]. We speculate that the different species of viruses might employ subtly different mechanisms to engage the activation of the STING signaling.

Functional analyses firmly established the essential role of SCAP in mediating the STING signaling. The phosphorylation of IRF3 stimulated by cytosolic DNAs is markedly impaired when knocking down the endogenous SCAP. Silencing the endogenous SCAP resulted in the impairment of the STING-mediated induction of IFNs and ISGs, and this effect is reversed by exogenously expressing siRNA-resistant SCAP. Silencing of *Scap* also impairs IFN-β protein production upon microbe infection, thus crippling the host antimicrobial responses against HSV-1 and *Listeria monocytogenes*. *In vivo* ‘knockdown’ of *Scap* induces less interferons and accelerates the death rate of the mice upon the HSV-1 infection.

We observed that HSV-1 infection triggers both SCAP and STING to traffic from the ER, via Golgi, to perinuclear microsome. Given that SCAP chaperons SREBP from ER to Golgi via the COP-II vesicle machinery, we had supposed that the translocation of STING is dependent on SCAP. However, knockdown of *Scap* did not affect the translocation of STING. Instead, the SCAP translocation is dependent on STING, but not on MAVS or TBK1, as evidenced in the corresponding knockout MEFs. The autophagy-related proteins (Atg9a and LC3) were implicated to modulate STING translocation [26]. It remains to address whether the autophagy proteins could modulate the action of SCAP.

Our study further provides a possible mechanism of regulating the STING signaling pathway by SCAP, which York *et al*. did not address. SCAP interacts individually with either STING or IRF3, via its N-terminal trans-membrane domains or C-terminal cytosolic domain respectively. The association between STING and IRF3 was respectively enhanced or impaired in response to HSV-1 stimulation, in the presence or absence of SCAP. Mis-targeting of SCAP, to whole cell, mitochondria or nucleus, resulted in its failure to bridge STING to IRF3. Notably, IRF3 also congregates to the perinuclear microsome after HSV-1 infection; and this congregation is dependent on SCAP, but not on TBK1. However, it is technologically challenging to demonstrate whether SCAP recruits IRF3 on ER or on perinuclear microsome. Recently, Dobbs et al. [39] have suggested that STING is retained on ER by some unknown inhibitor(s) in unstimulated cells. We think that such inhibitor(s) could not only block the translocation of STING/SCAP but also mask the IRF3 binding site on SCAP. Microbial infection releases this inhibition and triggers the translocation of STING and SCAP. Consequently, SCAP could recruit IRF3 to the STING signalosome. It is conceptually possible that IRF3 is blocked access to SCAP on ER by some “safe mechanism”, and SCAP on the perinuclear microsome is exposed to IRF3.

We had proposed an evolutionary necessity for assembling the STING signalosome on ER [27]. Arguably, the current characterization of SCAP further substantiates this perspective. We speculate that SCAP was dedicated primarily to mediate the activation of SREBP. Host innate immunity adapted it, along with AMFR and INSIG1, to integrate into the later-evolved STING signalosome. We predict that future studies will uncover more proteins essential for innate immunity that modulate the ERAD and/or Lipid/Glucose metabolism on ER. Deeper functional links between innate immunity and metabolism are expected to be displayed on the interface of ER.

Taken together, this study identified SCAP as the long-sought-after adaptor for recruiting IRF3 onto the STING signalosome. All the evidence favors the STING as an assembly platform, and the translocation of STING is the major cause of the other accompanying congregations. It remains to address what drives the STING translocation and whether the COP-II vesicle is required. The microenvironment of Golgi or microsome is probably favorable to the activation of TBK1 and IRF3. The underlying mechanisms remain elusive.

## Materials and Methods

### Ethics statement

C57BL/6 mice 6–8 weeks old were purchased from the Shanghai SLAC Laboratory Animal Company. The mice were maintained under specific pathogen-free (SPF) conditions at the Shanghai Institute of Biochemistry and Cell Biology. Animal experiments were carried out in strict accordance with the regulations in the Guide for the Care and Use of Laboratory Animals issued by the Ministry of Science and Technology of the People’s Republic of China. The protocol and the procedures for mice study were approved by the Institutional Animal Care and Use Committee of the Shanghai Institute of Biochemistry and Cell Biology, Chinese Academy of Sciences (Permit Number: IBCB0027 Rev2).

### Plasmids

SCAP, STING, TBK1, IRF3, INSIG1, cGAS, p65 were obtained by PCR from the thymus cDNA library and subsequently inserted into indicated mammalian expression vectors. The reporter plasmids (IFN-β-luciferase, pTK-Renilla) have been described previously [40]. The SCAP siRNA-resistant form was generated with silent mutations introduced into the siRNA target sequence. All point mutations were introduced by using a QuickChange XL site-directed mutagenesis method (Stratagene). All constructs were confirmed by sequencing.

### Antibodies and reagents

The polyclonal antibody against STING was generated by immunizing rabbit with recombinant human STING (221–379 aa). The goat polyclonal antibody against SCAP was from Santa Cruz Biotechnology. The rabbit polyclonal antibody against SCAP was a gift from Dr. Xiongzhong Ruan (Chongqing medical university). hemagglutinin (HA), Myc, Ub, SREBP1/2 and IRF3 antibody were purchased from Santa Cruz Biotechnology. TBK1 antibody was from abcam. Tom20 antibody was from BD Biosciences. Flag and β-actin antibodies were obtained from Sigma-Aldrich. Phospho-IRF3 and Phospho-TBK1 antibody was from Cell Signaling Technology.

Poly(dA:dT) and lipopolysaccharide (LPS) was obtained from Sigma-Aldrich. Poly(I:C) was purchased from Invitrogen. Wild type HSV-1 and HSV-1-GFP were kindly provided by Dr. Wentao Qiao (Nankai University) and Dr. Chunfu Zheng (Suzhou University), respectively. *Listeria monocytogenes* (10403 serotype) was a gift from Dr. Youcun Qian (Institute of Health Sciences). TBK1 kinase inhibitor BX795 was purchased from InvivoGen. ISD (Interferon stimulatory DNA) was prepared by annealing equimolar amounts of sense and antisense DNA oligonucleotides at 95°C for 10 min before cooling to room temperature. Oligonucleotides used are as follows:

ISD(sense), 5′-TAC AGA TCT ACT AGT GAT CTA TGA CTG ATC TGT ACA TGA TCT ACA-3′;
ISD (antisense), 5′-TGT AGA TCA TGT ACA GAT CAG TCA TAG ATC ACT AGT AGA TCT GTA-3′.

### Cell culture and transfection

HEK293, HEK293T and MEF cells were obtained from the American Type Culture Collection (ATCC). The procedure for generating BMDMs (bone marrow-derived macrophage) has been described previously [41]. HEK293, HEK293T, *Sting* ^*-/-*^ MEF, *Tbk1* ^*-/-*^ MEF and *Mavs* ^*-/-*^ MEF cells were cultured in DMEM medium (Invitrogen) plus 10% FBS and 1% penicillin-streptomycin (Invitrogen). Transfection was performed with Lipofectamine 2000 (Invitrogen) according to the manufacturer’s instructions.

### siRNA

The siRNAs duplexes were synthesized from GenePharma. The sequences of siRNAs are shown as follows:

*SCAP* siRNA1302, 5′-CCU ACC UUG UGG UGG UUA UTT-3′;
*Scap* siRNA 3060, 5′-GCT TAG AGC TGC AAG GCA ATT-3′;
*Scap* siRNA 3465, 5′-GAC UCG UGU UGC CUC UUU ATT-3′;
*Insig1*, 5′-GCA GUG AGU UGG AGG AAG ATT-3′;
*Srebp1*, *5′-GGU CUU CUA UCA AUG ACA ATT-3′;*
*Tom20*, *5′-CAA GUU ACC UGA UUU AAA A-3′*
The nonspecific siRNA (N.C.), 5′-UUC UCC GAA CGU GUC ACG UTT-3′.

### Immunofluorescence and confocal microscopy

MEF cells were grown on coverslips in 12-well plate. After treatment with or without HSV-1, coverslips with the cells were fixed for 15 minutes with 4% formaldehyde in PBS and permeabilized in 0.25% Triton X-100 in PBS for another 15 minutes, following by using 5% BSA in PBS for 1 hour. Then, cells were stained with indicated primary antibodies followed by incubation with fluorescent-conjugated secondary antibodies. The nuclei were counterstained with DAPI (Sigma-Aldrich). For mitochondria staining, living cells were incubated with 300 nM Mito Tracker Red (Invitrogen) for 30 min at 37°C. Slides were mounted with fluorescent mounting medium (Dako). Images were captured using a confocal microscope (TCS SP2 ACBS; Leica) with a ×63 (numerical aperture 1.4) oil objective.

### Immunoprecipitation assay and immunoblot analysis

For immunoprecipitation assay, cells extracts were prepared by using lysis buffer (50 mM Tris-HCl pH 7.4, 150 mM NaCl, 0.5% Triton X-100, 1mM EDTA) supplemented with a protease inhibitor cocktail (Roche). Lysate were incubated with appropriate antibodies for 4 hours to overnight at 4°C before adding protein A/G agarose beads for another 2 hours. The beads were washed three times with the lysis buffer and eluted with SDS-loading buffer by boiling for 5 minutes.

For immunoblot analysis, the immunoprecipitates samples were subjected to SDS-PAGE. The separated proteins were then electrically transferred to a PVDF membrane (Millipore). Immunoblotting was probed with indicated primary and secondary antibodies. The protein bands were visualized by using a SuperSignal West Pico chemiluminescence ECL kit (Pierce).

### Luciferase reporter assays

Luciferase reporter assays were performed as described previously [42].

### Real-time RT-PCR

Isolation of Total RNA from indicated cells was performed by using TRIzol reagent (Invitrogen) according to the manufacturer’s instructions. Reverse transcription of purified RNA was performed using oligo (dT) primers. The quantification of indicated gene transcripts were performed by real-time PCR with using FastStart Universal SYBR GREEN MASTER MIX (Roche), and *Gapdh* served as an internal control. PCR primers of indicated target genes are shown as below:

*Gapdh*: sense (5′-GAA GGG CTC ATG ACC ACA GT-3′),
  antisense (5′-GGA TGC AGG GAT GAT GTT CT-3′);
*Ifnb*: sense (5′- AGA TCA ACC TCA CCT ACA GG-3′),
  antisense (5′-TCA GAA ACA CTG TCT GCT GG-3′);
*Ifna4*: sense (5′- ACC CAC AGC CCA GAG AGT GAC C-3′),
  antisense (5′-AGG CCC TCT TGT TCC CGA GGT-3′);
*Cxcl10*: sense (5′- CGA TGA CGG GCC AGT GAG AAT G-3′),
  antisense (5′- TCA ACA CGT GGG CAG GAT AGG CT -3′);
*Scap*: sense (5′-TGC TGA AGC TCC CCT TGC CT-3′),
  antisense (5′-CAG AAG ATT TCT GTG CCA GG-3′);
Chop: sense (5′-CTG GAA GCC TGG TAT GAG GAT-3′),
  antisense (5′-CAG GGT CAA GAG TAG TGA AGG T-3′);
Erdj4: sense (5′-ATA AAA GCC CTG ATG CTG AAG C-3′),
  antisense (5′-GCC ATT GGT AAA AGC ACT GTG T -3′);
sXbp-1: sense (5′- CTG AGT CCG AAT CAG GTG CAG-3′),
  antisense (5′- GTC CAT GGG AAG ATG TTC TGG -3′);
tXbp-1: sense (5′- TGG CCG GGT CTG CTG AGT CCG -3′),
  antisense (5′- GTC CAT GGG AAG ATG TTC TGG -3′).

### Cell fractionation

Cell fraction was performed as described previously [23]. Cells were harvested by centrifugation at 1,000g for 10 min. The pellet was resuspended in sucrose homogenization buffer (0.25 M sucrose, 10 mM HEPES, pH 7.4), and cells were lysed by using a dounce homogenizer. Lysed cells were centrifuged at 500g for 10 min, and the supernatant was collected. The supernatant was then centrifuged at 10,300g for 10 min. The supernatant was crude microsomal (microsome and cytosol), and the pellet was crude mitochondrial (MAM and mitochondria). The crude microsomal fraction was subjected to ultracentrifugation at 100,000g for 60 min. The pellet was microsome fraction.

### Measurement of cytokines

Concentrations of the cytokines in culture supernatants were measured by ELISA kit (PBL Biomedical Laboratories) according to the manufacturer’s instructions.

### *In vivo* shRNA transfection and viral infection

The shRNA was delivered into C57BL/6 mice with JetPEI transfection reagent (PolyPlus Transfection, San Marcos, CA) according to the manufacturer’s instructions [43,44]. The shRNA plasmid and JetPEI was each diluted into 100ml of 5% glucose, then mixed and incubated for fifteen minutes at room temperature at a final N/P ratio of 8. Finally, the mixture (200ml) was injected into each mouse via tail vein.

For analysis of *in vivo* ‘knockdown’ efficiency, mice were euthanized after forty-eight hours of *in vivo* shRNA transfection. The mRNA or protein of SCAP was respectively checked in leukocyte from blood or extracts from livers.

The control or *Scap* ‘knockdown’ mice were infected intravenously with HSV-1. The viability of the infected mice was monitored for 7 days. The mouse serum was collected at six hours after infection to measure cytokine IFNβ production by ELISA.

The shRNA was designed targeting mouse *Scap* 5′- GCT TAG AGC TGC AAG GCA A -3′ sequence.

### Statistics

Student’s t-test was used for statistical analysis of two independent treatments. Mouse survival curve and statistics were analyzed with log-rank (Mantel-Cox) test. P values of less than 0.05 were considered to be statistically significant.

### Accession numbers

The GenBank (http://www.ncbi.nlm.nih.gov/Genbank) accession numbers for the genes and gene products discussed in this paper are:

SCAP (NM_012235.2, NP_036367.2); STING (NM_198282.3, NP_938023.1); TBK1 (NM_013254.3, NP_037386.1); IRF3 (NM_001197122.1, NP_001184051.1); INSIG1 (NM_005542.4, NP_005533.2); cGAS (NM_138441.2, NP_612450.2); p65 (NM_021975.3, NP_068810.3).

## Supporting Information

S1 Fig Silencing of SCAP displayed no effect on the ER stress signaling. The nonspecific control (N.C.) or *Scap* siRNA were transfected into MEF cells. Induction of *Chop*, *Erdj4*, spliced (s) and total (t) *Xbp-1* mRNAs was measured by quantitative PCR after TG (thapsigargin) treatment. Data are presented as means ± SD from three independent experiments. *p < 0.05; **p < 0.01.
(TIF)

S2 Fig Ectopic expression of SCAP could not activate IFN-β-luciferase reporters. (A and B) The indicated plasmids were transfected into HEK293 cells (A) or MEF cells (B) together with IFN-β-luciferase and pTK-Renilla reporter plasmids. Twenty-four hours after transfection, luciferase assays were performed.
(TIF)

S3 Fig SCAP modulates IRF3 activation. (A) The indicated siRNA were transfected into HEK293 cells together with pRDIII-I-luciferase and pTK-Renilla reporter plasmids. Twenty-four hours after transfection, cells were transfected again with cGAS, STING, TBK1 or IRF3-5D for twenty-four hours before luciferase assays were performed. (B and C) The nonspecific control (N.C.) or *Scap* siRNA were transfected into MEF cells. Forty-eight hours after transfection, cells were stimulated with poly(dA:dT) (B) or ISD (C) for indicated time periods, and cell extracts were analyzed for TBK1 phosphorylation. (D) HEK293T cells were transfected with N.C. or *SCAP* siRNA. Twenty-four hours later, Flag-tagged STING and Myc-tagged AMFR along with Ub were transfected into the knockdown cells. Cell lysates were subjected to immunoprecipitation with an anti-Flag antibody and immunoblotted with indicated antibodies.(E and F) MEF cells were transfected with N.C. or *Scap* siRNA. After stimulation with HSV-1, cell lysates were immunoprecipitated with an anti-STING antibody or normal lgG and immunoblotted with indicated antibodies.(G) MEF cells were transfected with N.C. or *Scap* siRNA. After stimulation with HSV-1, MEF cells were immunostained with an anti-TBK1 antibody and imaged by confocal microscopy. Scale bars represent 25μm. Data from (A) are presented as means ± SD from three independent experiments. *p < 0.05; **p < 0.01.
(TIF)

S4 Fig Silencing of SCAP markedly impaired the dimerization and nuclear translocation of IRF3. (A) The nonspecific control (N.C.), *Scap* siRNA or *Tom20* siRNA were transfected into MEF cells. Forty-eight hours after transfection, cells were stimulated with ISD, and cell extracts were analyzed for IRF3 dimerization by native PAGE. (B) The nonspecific control (N.C.), *Scap* siRNA or *Tom20* siRNA were transfected into MEF cells. Forty-eight hours after transfection, cells treated with ISD, were stained with the antibody against IRF3, and imaged by confocal microscopy. Scale bars represent 50 μm.
(TIF)

S5 Fig SCAP co-localized with STING and IRF3 upon HSV-1 infection. (A and B) MEF cells were infected with HSV-1 and then immunostained with indicated antibodies and imaged by confocal microscopy. Scale bars represent 25μm. (C) Immunoblot analysis of fractionation experiments of uninfected or HSV-1infected MEFs. MAM, mitochondria-associated ER membrane.
(TIF)

S6 Fig SCAP promotes IRF3 binding to STING. (A) Schematic diagram of SCAP and its truncation mutants (upper panel). SCAP-Myc or its mutants were individually transfected into HEK293T cells along with Flag-IRF3. The cell lysates were immunoprecipitated with an anti-Myc antibody and then immunoblotted with the indicated antibodies (lower panel). (B) Schematic diagram of IRF3 and its truncation mutants (upper panel). Flag-IRF3 or its mutants were individually transfected into HEK293T cells along with SCAP-Myc. The cell lysates were immunoprecipitated with an anti-Myc antibody and then immunoblotted with indicated antibodies (lower panel). (C) MEF cells were transfected with N.C. or *Scap* siRNA. After stimulation with HSV-1, cell lysates were immunoprecipitated with an anti-STING antibody or normal lgG and immunoblotted with indicated antibodies. (D) WT or *sting*-/- MEF were infected with HSV-1, and the cell lysate were immunoprecipitated with an anti-IRF3 antibody or normal lgG and immunoblotted with indicated antibodies.
(TIF)

S7 Fig NLS-SCAP failed to interact with IRF3. Myc-tagged SCAP or its mutants was transfected into HEK293T cells along with Flag-tagged IRF3. Twenty-four hours after transfection, cell lysates were immunoprecipitated with an anti-Myc antibody, and then immunoblotted with the indicated antibodies.
(TIF)

S8 Fig Loss of SCAP has no effect on the replication of NDV-GFP. NDV-GFP replication in HEK293 cells transfected with N.C. or *SCAP* siRNA was visualized by fluorescence microscopy.
(TIF)

S9 Fig STING signaling is physically and functionally distinct from SREBP signaling. (A) HEK293T cells were transfected with the negative control (N.C.) or *Srebp1* siRNA. Cell lysates were immunoblotted with the indicated antibodies. (B and C) The nonspecific control (N.C.) or *Srebp1* siRNA were transfected into MEF cells. Forty-eight hours after transfection, cells were stimulated with ISD (B) or infected with HSV-1 (C). Induction of *Ifnb* and *Ifna4* mRNA was measured by quantitative PCR. (D) Immunofluorescence microscopy of SREBP1 in MEFs infected with or without HSV-1. Scale bars represent 25μm. (E) MEF cells were transfected with N.C. or *Scap* siRNA and then rescued with the indicated siRNA-resistant SCAP constructs. After ISD stimulation, induction of *Ifnb* and *Ifna4* mRNA was measured by qPCR. (F) MEF cells were grown in DMEM with or without FBS for 8 hours, and stimulated with or without HSV-1, respectively. Induction of *Ifnb* mRNA was measured by quantitative PCR. Data from (B), (C), (E) and (F) are presented as means ± SD from three independent experiments. *p < 0.05; **p < 0.01.
(TIF)
