# Supplementary figures and images for: ER Adaptor SCAP Translocates and Recruits IRF3 to Perinuclear Microsome Induced by Cytosolic Microbial DNAs
Source: PLoS Pathog. 2016 Feb 22;12(2):e1005462. doi: 10.1371/journal.ppat.1005462 (PMC4762662; doi:10.1371/journal.ppat.1005462)

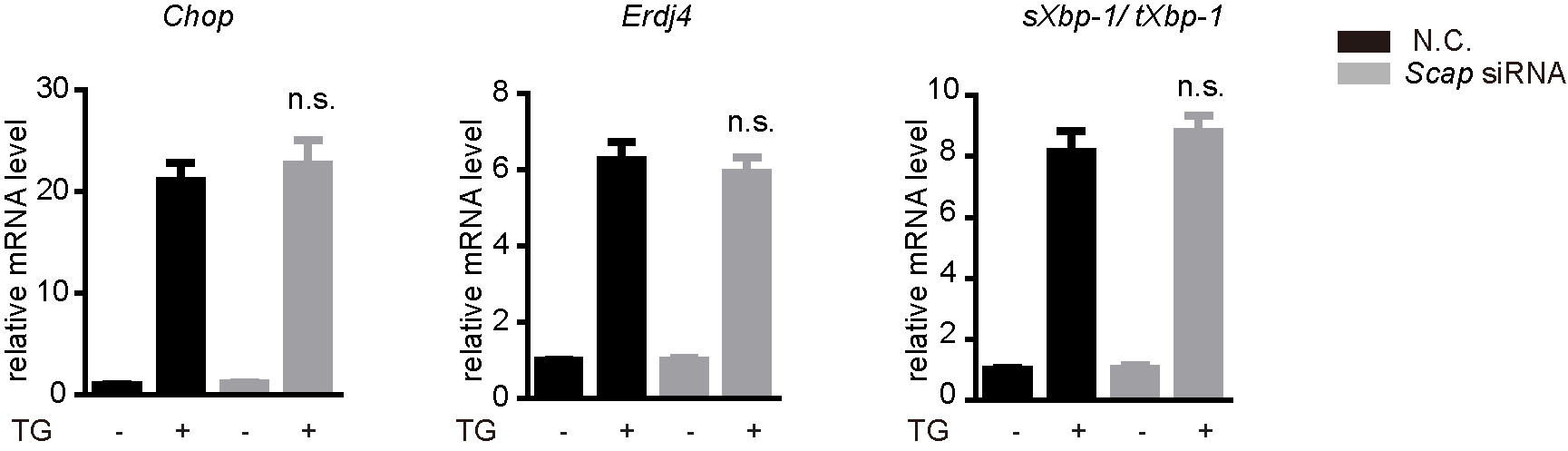

Supplement: S1 Fig — The nonspecific control (N.C.) or Scap siRNA were transfected into MEF cells. Induction of Chop, Erdj4, spliced (s) and total (t) Xbp-1 mRNAs was measured by quantitative PCR after TG (thapsigargin) treatment. Data are presented as means ± SD from three independent experiments. *p < 0.05; **p < 0.01. (TIF) [file ppat.1005462.s001.tif]

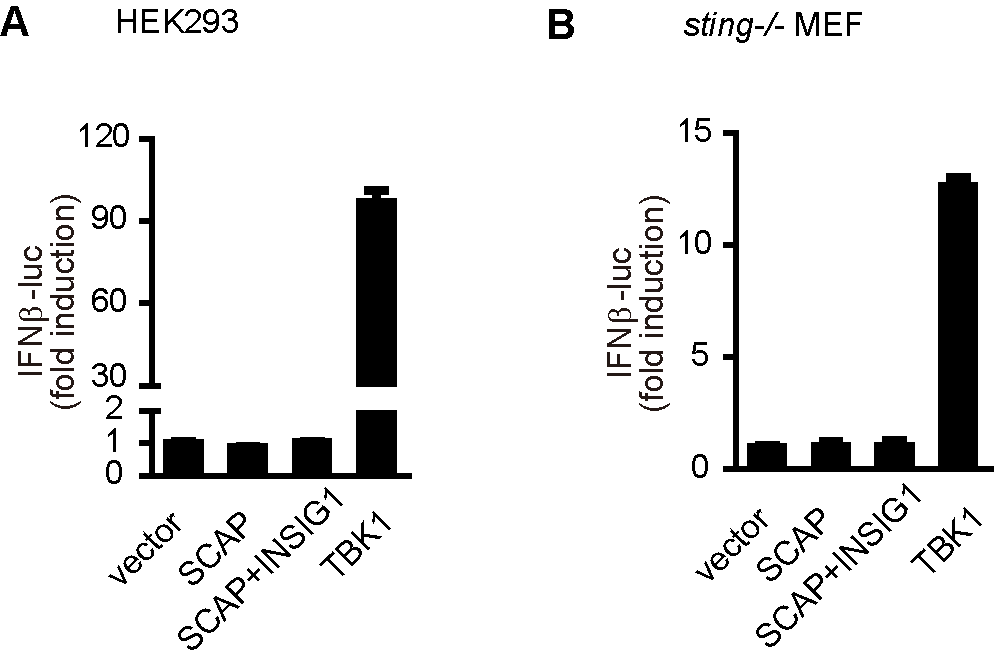

Supplement: S2 Fig — (A and B) The indicated plasmids were transfected into HEK293 cells (A) or MEF cells (B) together with IFN-β-luciferase and pTK-Renilla reporter plasmids. Twenty-four hours after transfection, luciferase assays were performed. (TIF) [file ppat.1005462.s002.tif]

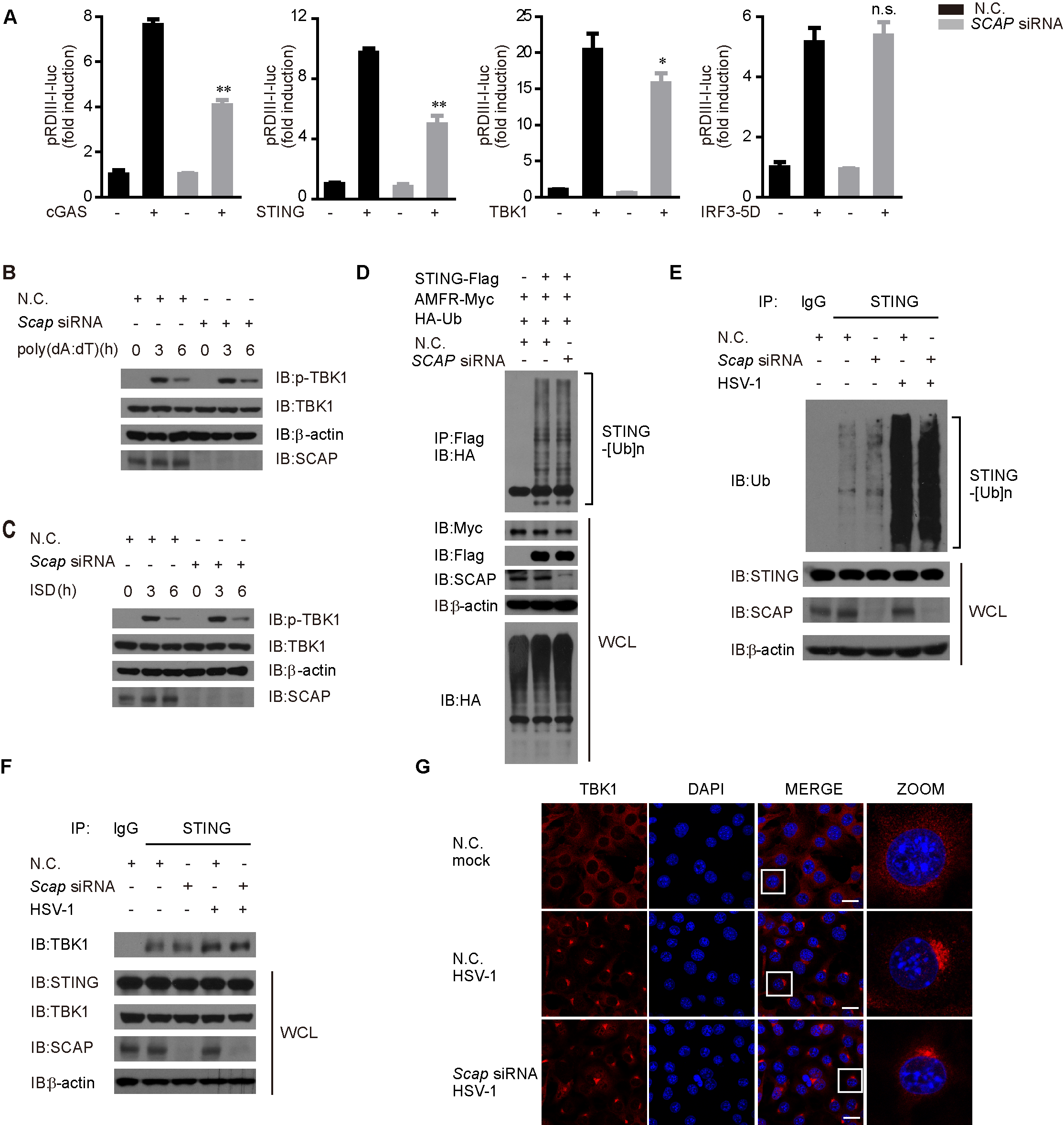

Supplement: S3 Fig — (A) The indicated siRNA were transfected into HEK293 cells together with pRDIII-I-luciferase and pTK-Renilla reporter plasmids. Twenty-four hours after transfection, cells were transfected again with cGAS, STING, TBK1 or IRF3-5D for twenty-four hours before luciferase assays were performed. (B and C) The nonspecific control (N.C.) or Scap siRNA were transfected into MEF cells. Forty-eight hours after transfection, cells were stimulated with poly(dA:dT) (B) or ISD (C) for indicated time periods, and cell extracts were analyzed for TBK1 phosphorylation. (D) HEK293T cells were transfected with N.C. or SCAP siRNA. Twenty-four hours later, Flag-tagged STING and Myc-tagged AMFR along with Ub were transfected into the knockdown cells. Cell lysates were subjected to immunoprecipitation with an anti-Flag antibody and immunoblotted with indicated antibodies.(E and F) MEF cells were transfected with N.C. or Scap siRNA. After stimulation with HSV-1, cell lysates were immunoprecipitated with an anti-STING antibody or normal lgG and immunoblotted with indicated antibodies.(G) MEF cells were transfected with N.C. or Scap siRNA. After stimulation with HSV-1, MEF cells were immunostained with an anti-TBK1 antibody and imaged by confocal microscopy. Scale bars represent 25μm. Data from (A) are presented as means ± SD from three independent experiments. *p < 0.05; **p < 0.01. (TIF) [file ppat.1005462.s003.tif]

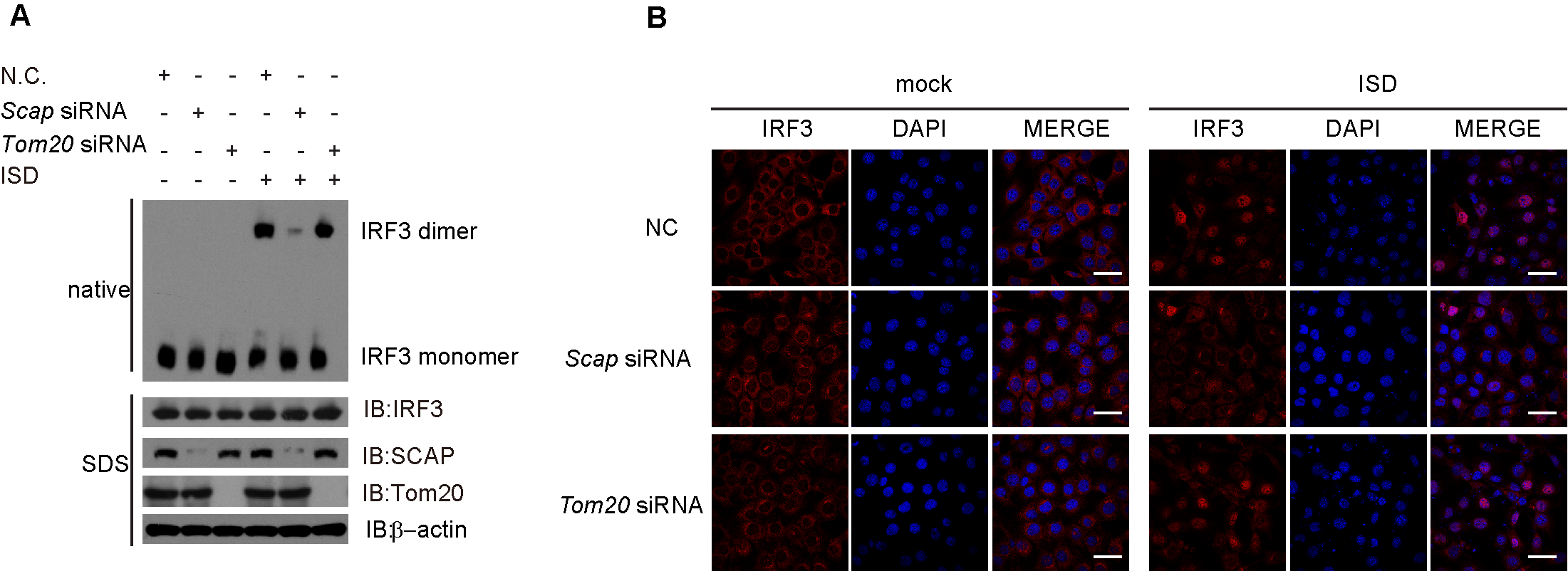

Supplement: S4 Fig — (A) The nonspecific control (N.C.), Scap siRNA or Tom20 siRNA were transfected into MEF cells. Forty-eight hours after transfection, cells were stimulated with ISD, and cell extracts were analyzed for IRF3 dimerization by native PAGE. (B) The nonspecific control (N.C.), Scap siRNA or Tom20 siRNA were transfected into MEF cells. Forty-eight hours after transfection, cells treated with ISD, were stained with the antibody against IRF3, and imaged by confocal microscopy. Scale bars represent 50 μm. (TIF) [file ppat.1005462.s004.tif]

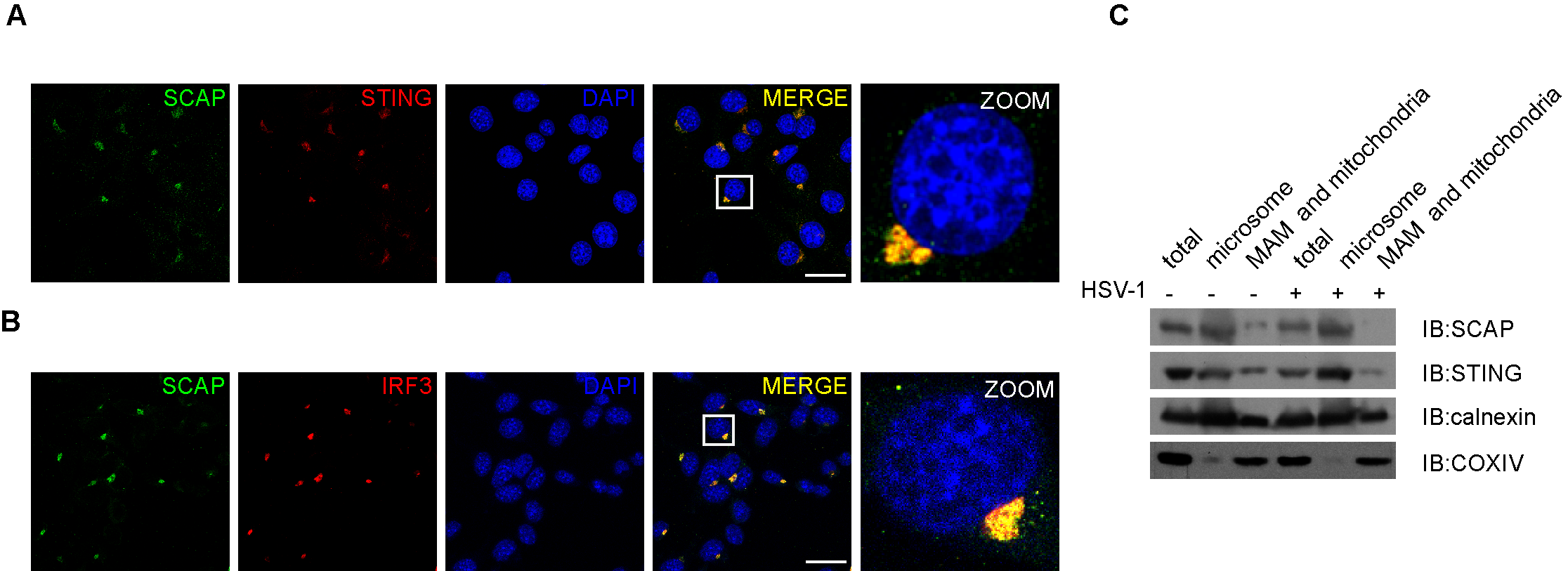

Supplement: S5 Fig — (A and B) MEF cells were infected with HSV-1 and then immunostained with indicated antibodies and imaged by confocal microscopy. Scale bars represent 25μm. (C) Immunoblot analysis of fractionation experiments of uninfected or HSV-1infected MEFs. MAM, mitochondria-associated ER membrane. (TIF) [file ppat.1005462.s005.tif]

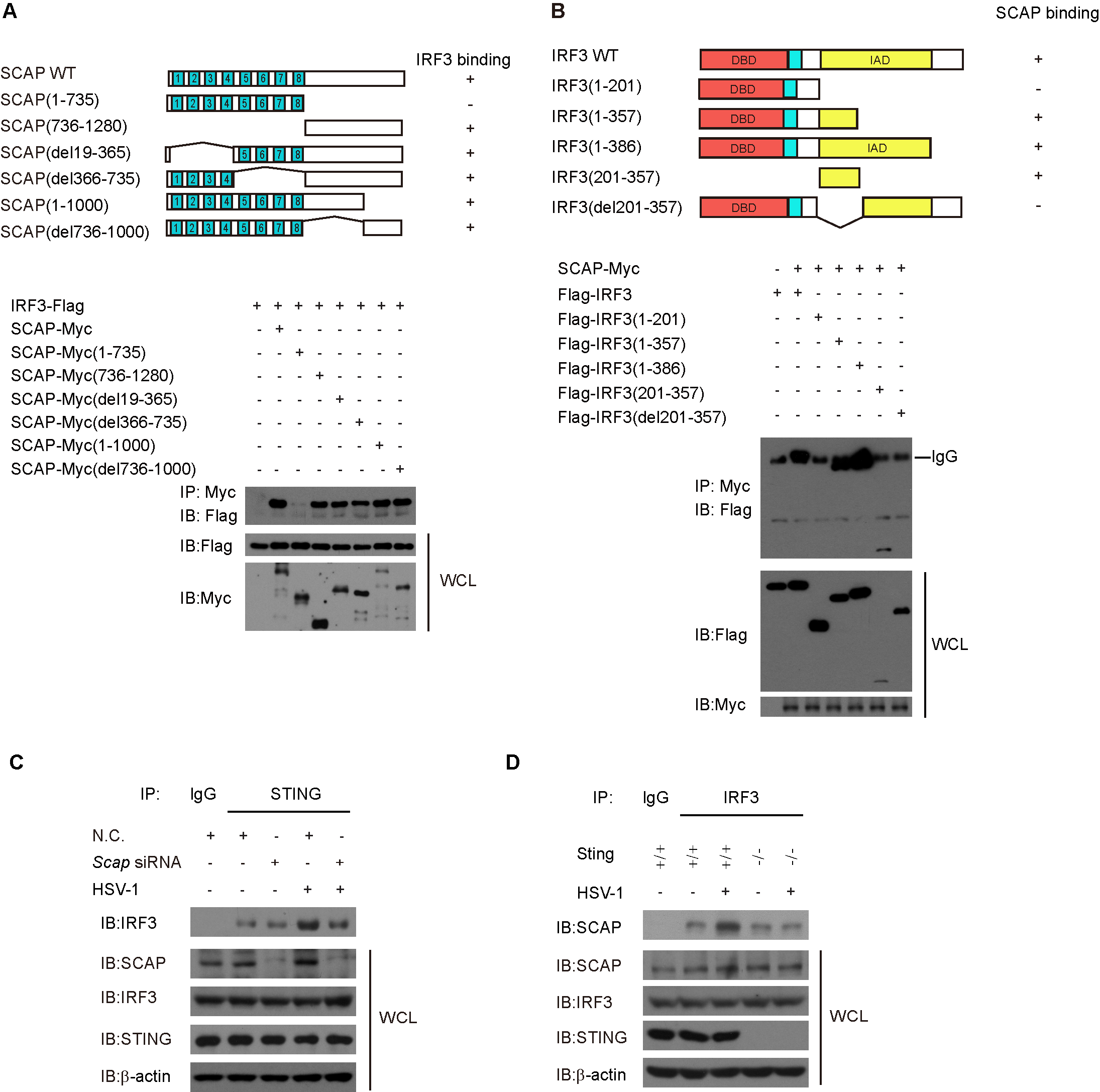

Supplement: S6 Fig — (A) Schematic diagram of SCAP and its truncation mutants (upper panel). SCAP-Myc or its mutants were individually transfected into HEK293T cells along with Flag-IRF3. The cell lysates were immunoprecipitated with an anti-Myc antibody and then immunoblotted with the indicated antibodies (lower panel). (B) Schematic diagram of IRF3 and its truncation mutants (upper panel). Flag-IRF3 or its mutants were individually transfected into HEK293T cells along with SCAP-Myc. The cell lysates were immunoprecipitated with an anti-Myc antibody and then immunoblotted with indicated antibodies (lower panel). (C) MEF cells were transfected with N.C. or Scap siRNA. After stimulation with HSV-1, cell lysates were immunoprecipitated with an anti-STING antibody or normal lgG and immunoblotted with indicated antibodies. (D) WT or sting-/- MEF were infected with HSV-1, and the cell lysate were immunoprecipitated with an anti-IRF3 antibody or normal lgG and immunoblotted with indicated antibodies. (TIF) [file ppat.1005462.s006.tif]

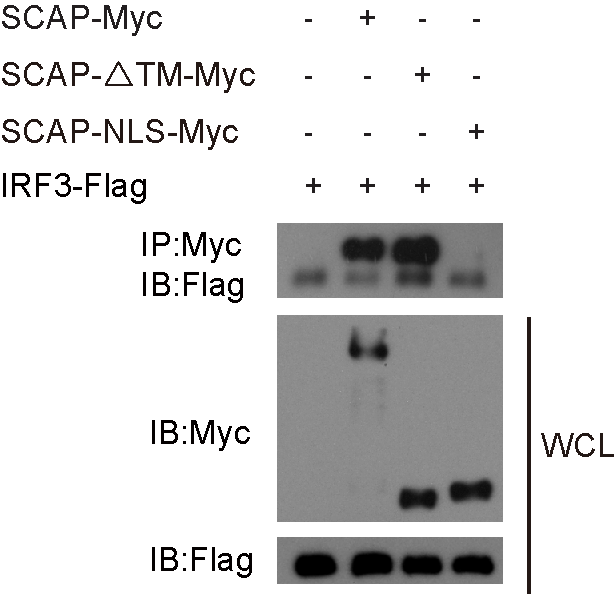

Supplement: S7 Fig — Myc-tagged SCAP or its mutants was transfected into HEK293T cells along with Flag-tagged IRF3. Twenty-four hours after transfection, cell lysates were immunoprecipitated with an anti-Myc antibody, and then immunoblotted with the indicated antibodies. (TIF) [file ppat.1005462.s007.tif]

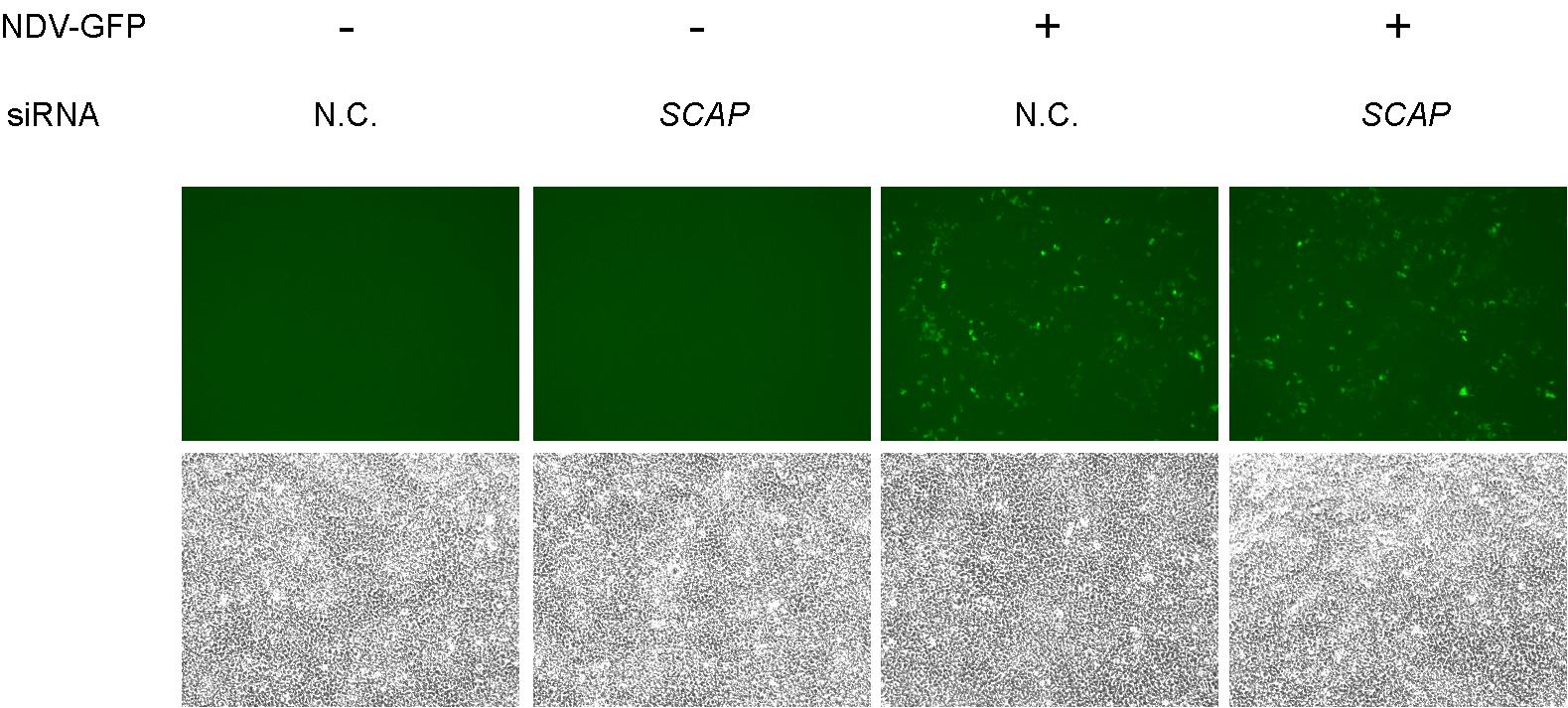

Supplement: S8 Fig — NDV-GFP replication in HEK293 cells transfected with N.C. or SCAP siRNA was visualized by fluorescence microscopy. (TIF) [file ppat.1005462.s008.tif]

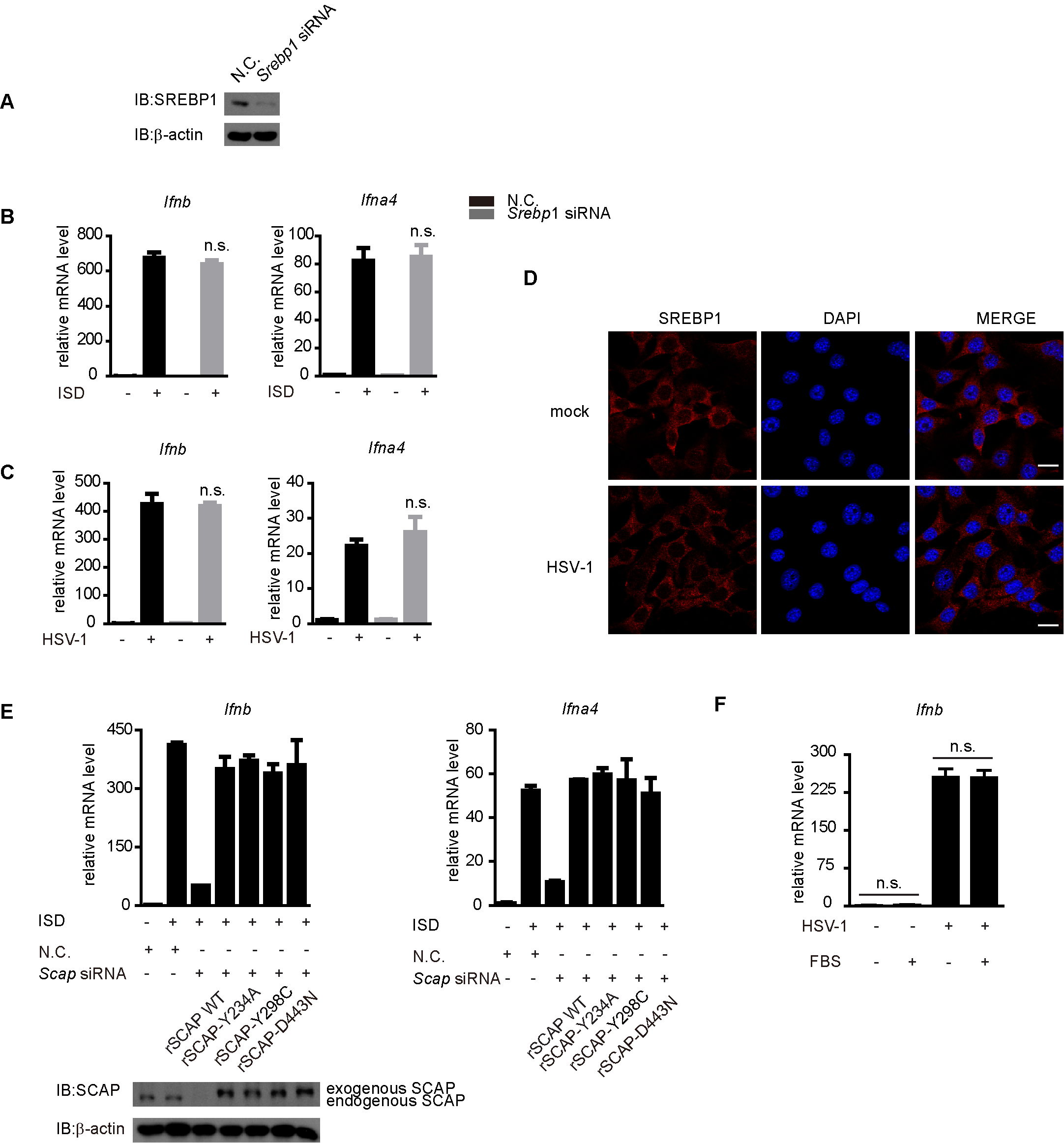

Supplement: S9 Fig — (A) HEK293T cells were transfected with the negative control (N.C.) or Srebp1 siRNA. Cell lysates were immunoblotted with the indicated antibodies. (B and C) The nonspecific control (N.C.) or Srebp1 siRNA were transfected into MEF cells. Forty-eight hours after transfection, cells were stimulated with ISD (B) or infected with HSV-1 (C). Induction of Ifnb and Ifna4 mRNA was measured by quantitative PCR. (D) Immunofluorescence microscopy of SREBP1 in MEFs infected with or without HSV-1. Scale bars represent 25μm. (E) MEF cells were transfected with N.C. or Scap siRNA and then rescued with the indicated siRNA-resistant SCAP constructs. After ISD stimulation, induction of Ifnb and Ifna4 mRNA was measured by qPCR. (F) MEF cells were grown in DMEM with or without FBS for 8 hours, and stimulated with or without HSV-1, respectively. Induction of Ifnb mRNA was measured by quantitative PCR. Data from (B), (C), (E) and (F) are presented as means ± SD from three independent experiments. *p < 0.05; **p < 0.01. (TIF) [file ppat.1005462.s009.tif]
